# Lateral habenula and periaqueductal gray neurons signal reward prediction error and continuity of reward expectancy to drive reward-seeking behavior

**DOI:** 10.1016/j.celrep.2025.116907

**Published:** 2026-01-23

**Authors:** Hyunchan Lee, Okihide Hikosaka

**Affiliations:** 1 Laboratory of Sensorimotor Research, National Eye Institute, National Institutes of Health, Bethesda, MD 20892-4435, USA; 2 Lead contact

## Abstract

Reward-seeking behaviors often require not only encoding moment-to-moment reward prediction errors but also sustaining reward expectancy in the face of repeated negative outcomes and ongoing effort costs. While the lateral habenula has been extensively studied as a source of negative prediction error signals, how the brain maintains motivational continuity when rewards are delayed, uncertain, or repeatedly omitted remains poorly understood. Here, we show a complementary role of the periaqueductal gray in sustaining reward expectancy through tonic activity that persists beyond prediction errors. We find that the balance between distinct tonic signaling in the periaqueductal gray, which signals remaining reward expectancy, and phasic signaling in the lateral habenula, which signals reward prediction error, plays a crucial role in determining whether animals continue or discontinue reward-seeking behaviors when encountering unexpected negative events. This mechanism is essential for efficiently navigating complex environments with diverse reward volatilities and ultimately contributes to maximizing reward acquisition.

## In brief

Lee and Hikosaka show how animals overcome challenges and ultimately achieve goals. They find that LHb and PAG neurons encode prediction errors and the continuity of reward expectancy, with tonic PAG activity sustaining reward expectancy despite prediction errors.

## Graphical abstract

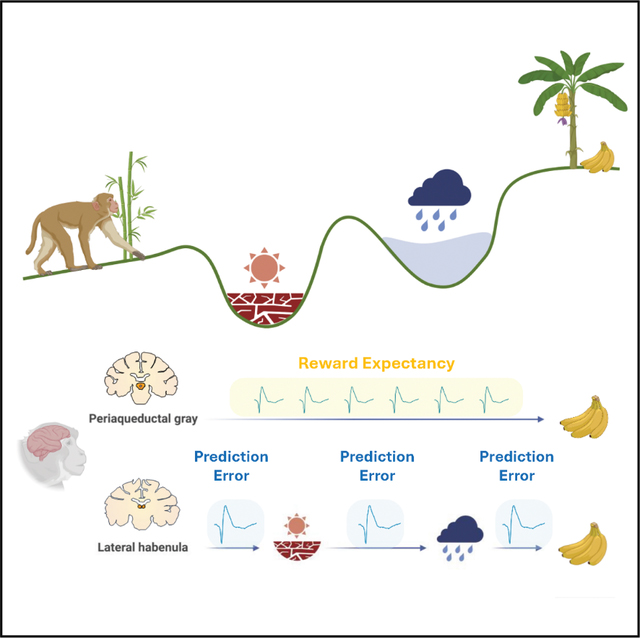

## INTRODUCTION

Goal-directed behavior in real-world environments often requires continuing actions despite repeated negative prediction errors, delayed rewards, and increasing effort demands.^1^ Although prediction error signaling, particularly in dopamine neurons, is known to shape learning and behavioral adjustment, it remains unclear how neural circuits sustain reward expectancy and motivation under such adverse conditions.^2,3^ Prediction error could be a crucial factor, for example, in environments where resources become scarce, helping animals adjust their behavioral patterns to minimize unnecessary energy expenditure (e.g., hibernation) or migrate to environments with richer resources.^4–9^ However, in real life, we often need to endure a series of unexpected events, some of which may be disappointing or irrelevant to immediate gratification, to ultimately achieve our desired outcomes.^10^ Thus, we hypothesized that there would be another important neuronal mechanism that signals the remaining reward expectancy, helping us overcome events that induced negative reward prediction error and continue motivated behaviors until we reach our desired goals.^11–13^ Then, how does the animal brain signal the prediction error and the remaining reward expectancy when encountering an event-induced negative reward prediction error? Understanding the balance between negative reward prediction errors and the persistence of motivated behaviors is key to uncovering the neural basis of cognitive flexibility and stable habits that support critical neurocognitive functions, such as social bonding and motor skills, and to understanding how this balance breaks down in disorders such as Parkinson’s disease.^14,15^

To solve this question, we examined the lateral habenula (LHb) and periaqueductal gray (PAG), key brain regions involved in the regulation of negative mood in animals.^16–18^ The LHb is a well-known primary input to dopamine neurons, especially signaling reward prediction error^19^ and aversiveness^20^ from stimuli. Thus, dysfunction of the LHb has been extensively studied as a promising therapeutic target for treating major depressive disorder.^21–24^ In the present study, we raised a question regarding a feature of the LHb response, which primarily involves phasic firing lasting 100–500 ms.^2^ This rapid signaling is crucial for animals to quickly learn and identify significant objects in a complex environment, where various valued objects are presented sequentially, and to promptly guide their actions step by step accordingly.^25–28^ However, if an animal’s reward-seeking behavior, which involves a series of sequential actions, is solely regulated by the phasic signaling of the LHb, the animal would easily abandon or leave the ongoing motivated behavior every time it encounters an event-induced negative reward prediction error.

Therefore, we investigated another key brain region involved in the regulation of negative moods: the PAG.^29–31^ Notably, PAG activity has primarily been reported to play a pivotal role in controlling coping strategies in response to aversive stimuli, such as defensive and escape behaviors.^32^ As a result, the PAG has been suggested to be a critical brain area that can provide therapeutic interventions not only for mental disorders^33–35^ but also for movement disorders related to the control of involuntary behaviors.^36–41^ This has been further supported by previous studies showing that PAG manipulation affects pain, analgesia,^42^ uncontrollable freezing behaviors,^43^ and involuntary vocalizations.^44^ Moreover, the PAG has crucial efferents that project to the motor control system, such as the raphe interpositus, which regulates gaze fixation in primates.^45^ Hence, we could expect that the PAG would not only trigger escape behaviors but would also play a critical role in controlling escape and sustaining reward-seeking behaviors when an animal encounters an event-induced negative reward prediction error.

In summary, we investigated how the LHb and PAG neurons signal prediction error and the remaining reward expectancy to understand how an animal decides whether to overcome or leave an event-induced negative reward prediction error. As a result, we found a significant correlation between PAG activity and the continuity of reward-seeking behavior in animals, which could help them overcome event-induced negative reward prediction error and ultimately obtain their desired rewards.

## RESULTS

**Figure 1.**
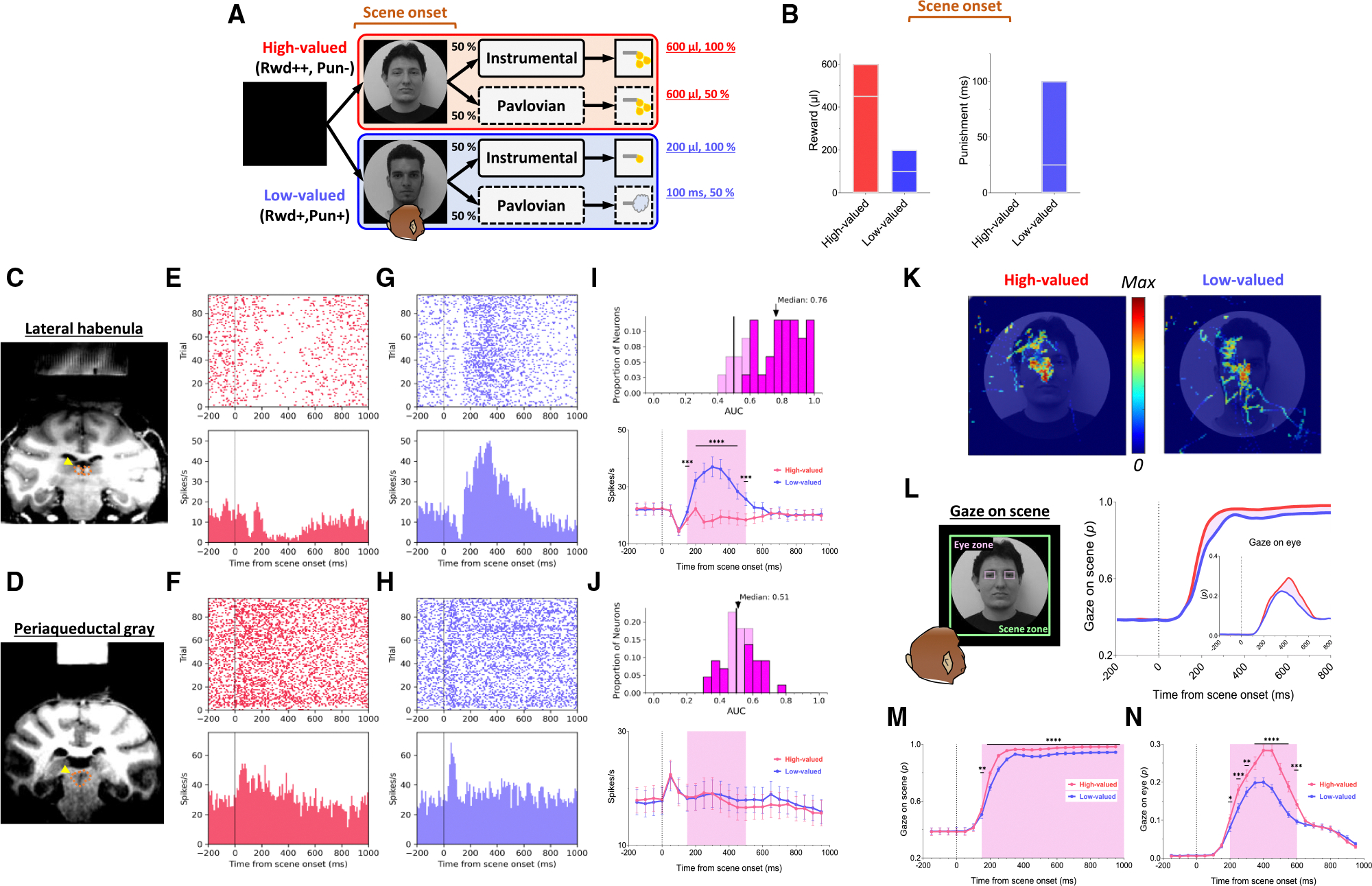
Neuronal responses and anticipatory gazes in scene viewing (A) In the scene-based instrumental/Pavlovian task, each trial began with the free viewing of a scene image. Each group of scene images provided respective reward outcomes from the instrumental and Pavlovian tasks. Consequently, the monkeys freely viewed each group of scene images based on the average rewards they had experienced during both tasks. (B) Bars indicate the maximum and minimum values of predictive reward and punishment outcomes before scene onset at the beginning of each trial, whereas midlines represent the mean amounts of juice received as a reward and of airpuff punishment calculated for high-valued and low-valued scene groups based on data combined from the instrumental and Pavlovian tasks. (C and D) Recording sites are marked with yellow arrowheads and orange dotted lines, indicating the locations of the LHb and PAG. (E–H) Raster plots and histograms of example LHb and PAG neurons, aligned to scene onset, with a 10 ms bin width. Red raster plots and histograms indicate neuronal activities during high-valued scenes (E and F), whereas blue raster plots and histograms indicate neuronal activities during low-valued scenes (G and H). (I and J) Top, area under the ROC curve (AUC) distributions. Bottom, average firing rates of LHb and PAG neurons calculated with a 50 ms bin width. Statistical differences between groups were analyzed using repeated-measures two-way ANOVA (LHb, main effect of group: F(1.000, 33.00) = 34.37, *p* < 0.0001; main effect of time: F(2.837, 93.61) = 14.32, *p* < 0.0001; interaction: F(2.103, 69.40) = 25.89, *p* < 0.0001, *n* = 34; and PAG, main effect of group: F(1.000, 43.00) = 1.295, *p* = 0.2614; main effect of time: F(2.178, 93.63) = 2.993, *p* = 0.0506; interaction: F(6.994, 300.7) = 2.108, *p* = 0.0427, *n* = 44), followed by Holm-Šídák’s multiple comparisons test (***p < 0.001 and ****p < 0.0001). The color-shaded areas represent the time window during which the phasic responses of LHb neurons showed significant differences between high-valued and low-valued scene images (150–500 ms). The AUC distributions for both LHb and PAG neurons were analyzed within this time window. Dark shading in the AUC distributions indicates the proportion of neurons showing significant differences between conditions. (K) An example heatmap illustrating monkeys’ gaze on face scene images. (L) Outside, the probabilities of gaze on face scene images during the free-viewing period (scene zone, 40° × 40°). Inside, the probabilities of gaze on the eye regions of face scene images during the free-viewing period (eye zone, 4° × 3°). (M and N) Averaged probabilities of gaze directed toward face scene images and eye regions, calculated using a 50 ms bin width. Statistical differences between groups were analyzed using repeated-measures two-way ANOVA (face, main effect of group: F(1.000, 71.00) = 42.98, *p* < 0.0001; main effect of time: F(1.333, 94.65) = 430.2, *p* < 0.0001; interaction: F(3.306, 234.8) = 13.30, *p* < 0.0001; and eye, main effect of group: F(1.000, 71.00) = 59.55, *p* < 0.0001; main effect of time: F(2.602, 184.7) = 139.5, *p* < 0.0001; interaction: F(4.089, 290.3) = 25.62, *p* < 0.0001, *n* = 72), followed by Holm-Šídák’s multiple comparisons test (*p < 0.05, **p < 0.01, ***p < 0.001, and ****p < 0.0001). The color-shaded areas represent the time window during which face viewing (150–950 ms) and eye contact (200–600 ms) exhibited significant differences between high-valued and low-valued scene images. Data are presented as the mean ± SEM.

In this study, we devised a scene-based instrumental/Pavlovian task and recorded neuronal and behavioral responses of rhesus macaque monkeys while they passed through a series of negative reward prediction errors to ultimately obtain their desired reward outcome. This task includes two distinct instrumental and Pavlovian tasks, performed on a shared background scene image (Figure 1A). As a result, the monkeys were able to predict different reward outcomes for each group of scene images based on the average reward outcomes experienced in the instrumental and Pavlovian tasks. Furthermore, the monkeys experienced multiple changes in reward predictions throughout the task, depending on the appearance of each group of scene images and the distinct tasks.

### Reward expectation facilitates visual attention to contexts

**Figure 2.**
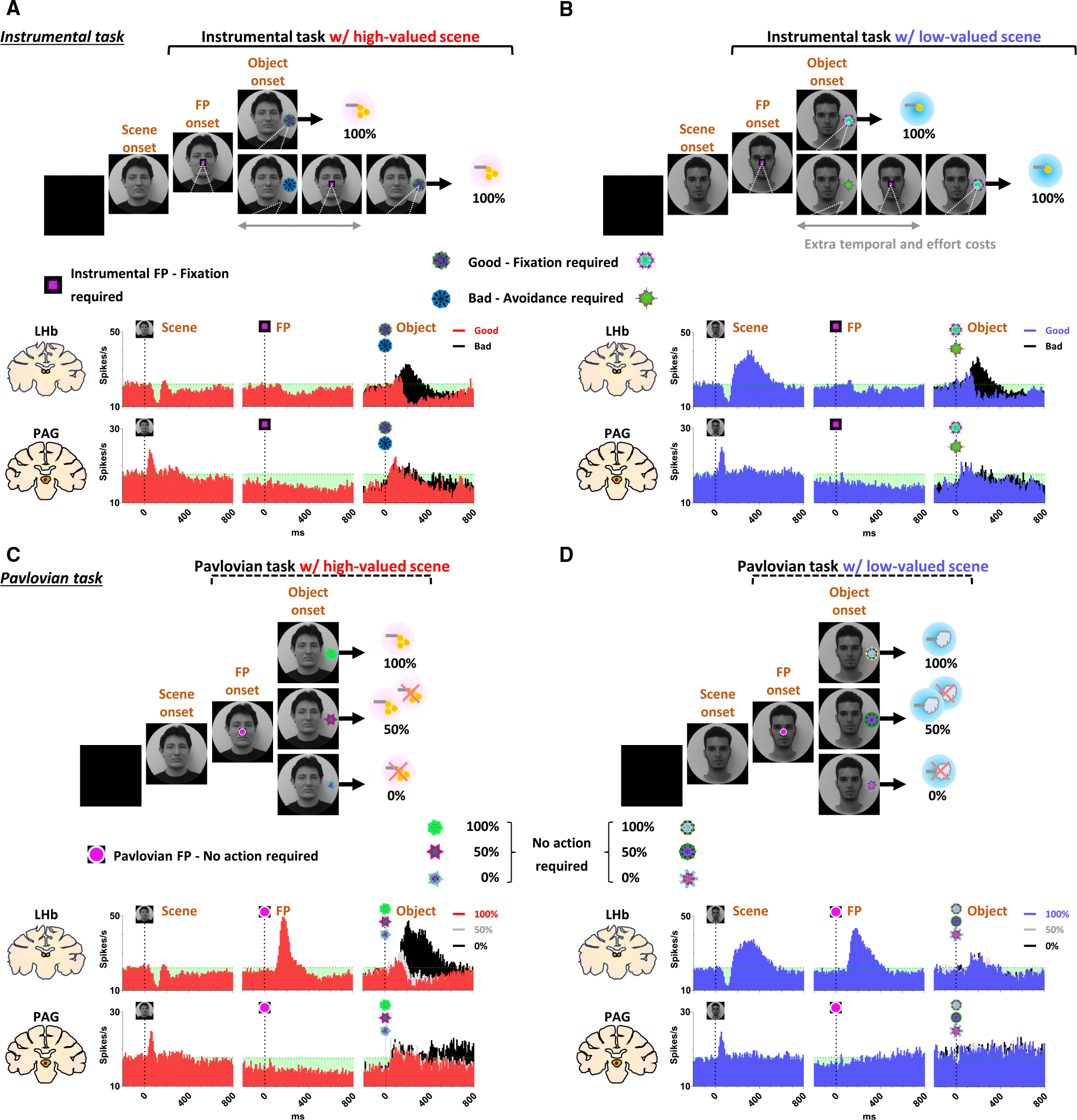
Neuronal and behavioral responses in instrumental and Pavlovian tasks (A) Histograms (10 ms bins) show the averaged neuronal activity in the LHb (top, *n* = 34) and PAG (bottom, *n* = 44) during the instrumental task with high-valued scenes. The histograms are aligned to the onset of each stimulus during the task procedures (scene onset, FP onset, and object onset). The green dotted line indicates baseline neuronal activity measured for 200 ms before scene onset. The green-shaded areas represent changes in neuronal activity compared to baseline levels during the task procedures (LHb, 22.13 spikes/s; PAG, 17.71 spikes/s). (B) The histograms show the same data as in (A) but for low-valued scenes. (C and D) The histograms show the same data as in (A) and (B), respectively, but for the Pavlovian task.

We first found that reward experiences facilitate the visual attention of primates. Each trial of the task started with the appearance of a scene image (Figure 1A, scene onset), which allowed the monkey’s free viewing for 1 s and then remained as a background scene until the end of the trial, during which either the instrumental or Pavlovian task was performed. The monkeys received a large amount of juice as a reward in both instrumental and Pavlovian tasks in the high-valued scenes (Figures 2A and 2C), while they received a small amount of juice as a reward in the instrumental task and airpuff punishments in the Pavlovian task in the low-valued scenes (Figures 2B and 2D). As a result, at the start of a trial, monkeys could predict greater reward outcomes from the appearance of the high-valued scenes compared to low-valued scenes (Figure 1B).

As the monkeys experienced larger rewards, their free viewing became more focused on high-valued scenes (Figure 1L). For the background scene images, we used face and landscape images, as they are representative examples of social and spatial contexts that animals encounter during reward-seeking behaviors in real life.^46^ The data for face scene images only are presented in Figure S1, and the data for landscape scene images only are shown in Figure S2. As shown in these figures, the LHb and PAG exhibited consistent neuronal response patterns across both face and landscape scene images. Gaze patterns, including scene gaze and anticipatory gaze, were also stronger for high-valued scenes than for low-valued scenes in both face and landscape scene images. As the neuronal and behavioral responses were consistent across face and landscape scene images, we used the averaged responses of those in the main figures. When we used face images as the background scene images, they showed more pronounced gazes toward the eye regions in the high-valued face scene images than in the low-valued scene images, accompanied by stronger scene viewing of the high-valued face images (Figures 1M and 1N). The scene zone for gaze analysis was defined to match the diameter of the image (40°).

Through an additional experiment in which the influence of reward and punishment on scene gaze was examined respectively, we confirmed that the effect of value on monkeys’ gaze for scene images was particularly regulated by the predicted values of reward rather than airpuff punishment in this task design (Figures S1 and S2). The monkeys’ gaze toward the scene images was more focused on those with expected high-value rewards, even if they included an airpuff punishment, and it decreased for images with expected low-value rewards, even if there was no airpuff punishment.

### Continuity of reward expectancy facilitates subsequent actions even after negative reward prediction error

We then found that the reward expectancy from the scene images continuously facilitated the subsequent actions of animals in the next step. As described above, each trial began with the appearance of a scene image and the monkeys’ free viewing for 1 s (Figure 1A). In the meantime, although the monkeys’ gaze was more focused on the high-valued scenes than on the low-valued scenes, we found that their gaze toward the scene images commonly increased during the 50–150 ms period for both scenes, reaching over 90% and being sustained until the next stimulus appeared (Figure 1M). At the onset of the low-valued scenes, the monkeys sometimes closed their eyes or shifted their gaze away from the scene image during the free-viewing period. However, they soon returned their gaze to the scene and prepared to perform the subsequent task. This indicates that the monkeys had a strong motivational engagement for the subsequent task procedures in both scene groups. Notably, these highly motivated states in both scenes consistently facilitated subsequent behaviors.

**Figure 3.**
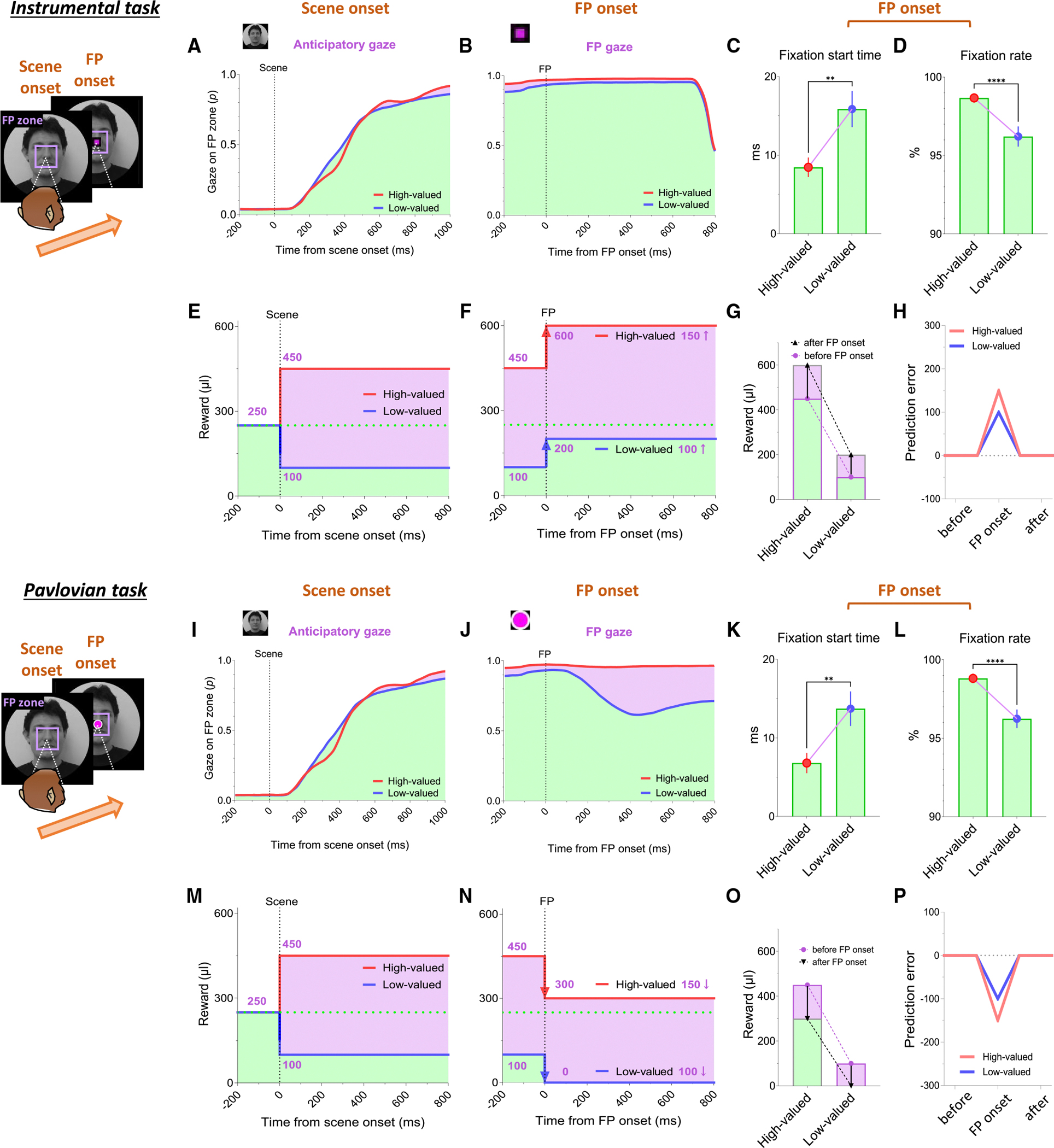
Reward expectancy facilitates subsequent actions (A) The probabilities of anticipatory gaze before the FP onset, where the gaze was located at the position where the FP would appear (FP zone, 10° × 10°). The purple-shaded areas represent differences in responses between high-valued and low-valued scenes or between good objects and bad objects. The green-shaded areas indicate the monkeys’ gaze on the location where the FP appeared, starting from scene onset and maintained throughout the subsequent task procedures. The purple-shaded areas highlight the differences in gaze responses between high-valued and low-valued scenes. (B) The probabilities of gaze on the FP after its appearance. (C) The fixation start time, representing the gaze-reaching time for monkeys to initiate fixation on the FP (high valued, 8.454 ± 1.244; low valued, 15.87 ± 2.293; Wilcoxon matched-pairs signed rank test; **p < 0.01, *n* = 72). (D) The fixation rate, indicating the percentage of time monkeys maintained their gaze on the FP to engage in the next task procedure (high valued, 98.67 ± 0.2923; low valued, 96.21 ± 0.6428; Wilcoxon matched-pairs signed rank test; ****p < 0.0001, *n* = 72). (E and F) Theoretical changes in predictive reward values from scene onset to FP onset in the instrumental task. (G) Theoretical changes in predictive reward values at FP onset in the instrumental task. The purple-shaded area indicates the difference in predictive reward values before versus after FP onset, and the green-shaded area indicates remaining predictive reward values after FP onset. (H) Prediction error values induced by FP onset, representing the difference in predictive reward values before versus after FP onset. (I and J) Same as (A) and (B) but in the Pavlovian task. </p/> (K and L) Same as (C) and (D) but in the Pavlovian task procedure (fixation start time: high valued, 6.801 ± 1.288; low valued, 13.72 ± 2.196; and fixation rate: high valued, 98.81 ± 0.2967; low valued, 96.24 ± 0.5895; Wilcoxon matched-pairs signed rank test; **p < 0.01 and ****p < 0.0001, *n* = 72). (M and N) Same as (E) and (F) but in the Pavlovian task. (O and P) Same as (G) and (H) but in the Pavlovian task. Data are presented as the mean ± SEM.

After a free viewing of the scene for 1 s, a fixation point (FP) appeared at the center of the screen, and either the instrumental task or the Pavlovian task was initiated (Figure 2). The instrumental task and the Pavlovian task were distinguished by the different shapes of the FP (Figures 2A and 2B, instrumental task, square; Figures 2C and 2D, Pavlovian task, circle). Once the FP appeared at the center of the scenes, the monkeys quickly fixated their gaze on the FP within 20 ms, whether it indicated the instrumental task or the Pavlovian task (Figures 3C and 3K). In both instrumental and Pavlovian tasks, the start time at which monkeys’ gaze reached the FP was significantly quicker in high-valued scenes than in low-valued scenes. In addition to the speed of the fixation start time, the monkeys exhibited higher fixation rates in high-valued scenes than in low-valued scenes (Figures 3D and 3L). However, even in low-valued scenes, monkeys initiated fixation on the FP within 20 ms (Figures 3C and 3K, blue), and the fixation rates were over 95% (Figures 3D and 3L, blue). This implies that the monkeys were in a strong motivational state in both high-valued and low-valued scenes. Indeed, even before the FP onset, about 90% of the monkeys’ gazes had already stayed on the location where the FP would appear (Figures 3A, 3B, 3I, and 3J).

This result implies that as long as reward expectancy is sustained (Figures 3M–3O, high valued), animals can maintain a strong motivational state (Figures 3I–3L, high valued) even after negative reward prediction error (Figure 3P, high valued). How, then, do the neural mechanisms in the brain operate to sustain such a strong motivational state in animals, even after the negative reward prediction error?

### PAG neurons signal reward expectancy using tonic activity

We found that the neuronal activity of the PAG was tonically modulated by the expectancy of reward outcomes. Along with the monkeys’ gaze behaviors, we recorded neuronal activities in the LHb and PAG (Figures 1C and 1D). When a scene image appeared at the beginning of a task trial, neuronal activity in both brain regions was inhibited by high-valued scenes and excited by low-valued scenes (Figures 1E–1J). However, their responses exhibited distinct characteristics, with the PAG primarily showing tonic firing, while the LHb responded with phasic firing. We notably found that the reward expectancy induced by the scene onset had a significant and continuous effect not only on the continuity of the monkeys’ subsequent scene-viewing behaviors (Figure 1M) but also on the tonic activity of PAG neurons, which persisted from scene onset to FP onset (Figure 1J).

**Figure 4.**
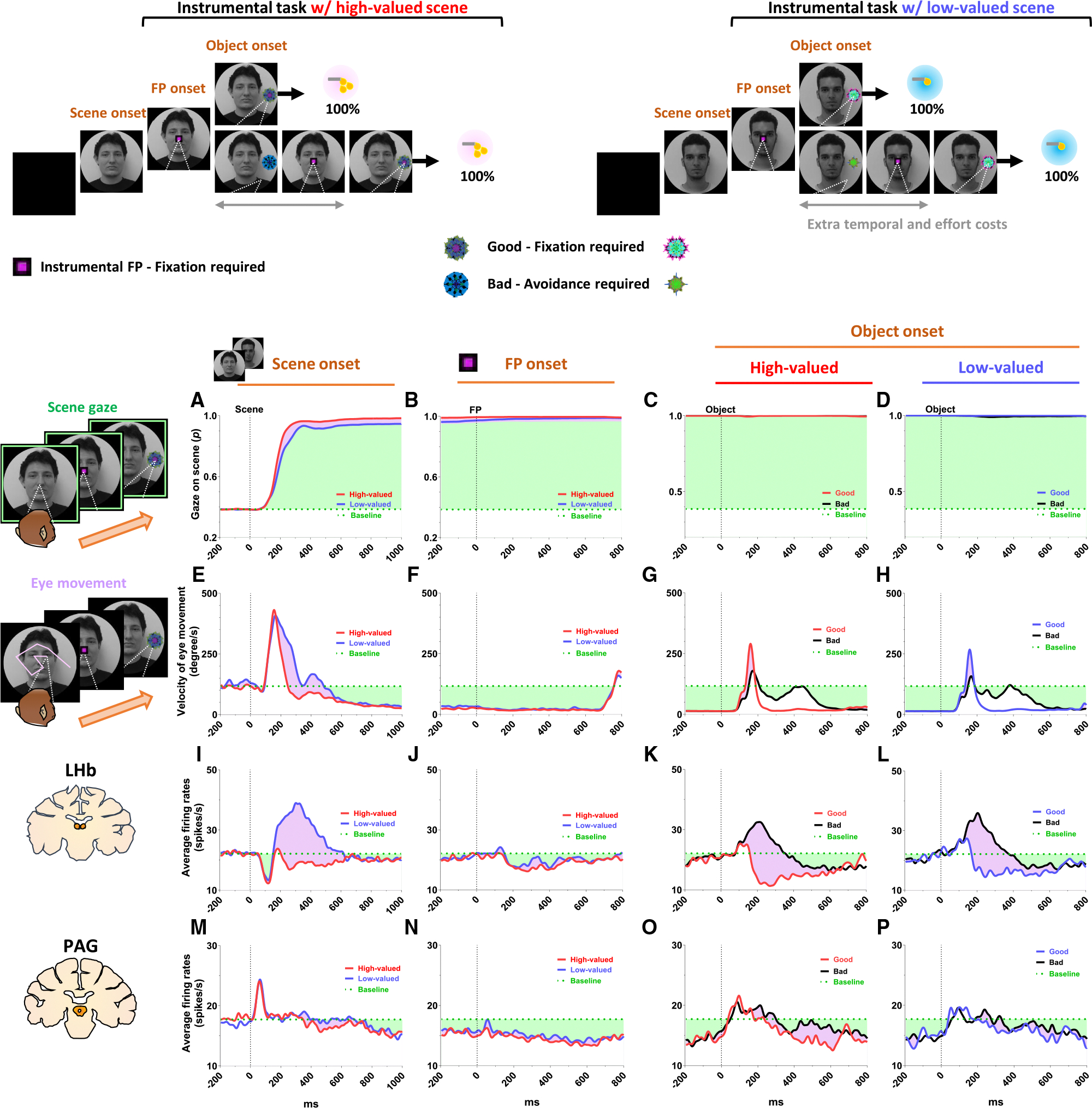
Tonic inhibition of PAG signals continuity of reward expectancy in an instrumental task (A–D) The probabilities of gaze fixation on scene images during the instrumental task. The purple-shaded areas represent differences in responses between high-valued and low-valued scenes or between 100% objects and 0% objects. The green dotted line indicates baseline levels measured for 200 ms before scene onset, while the green-shaded areas represent changes in gaze responses relative to baseline during the task procedures. (E–H) Same as (A)–(D) but for the velocity of eye movement. (I–L) The LHb activity shown in Figures 2A and 2B aligned with the gaze response presented in (A)–(H). (M–P) The PAG activity corresponds to the data presented in (I)–(L).

**Figure 5.**
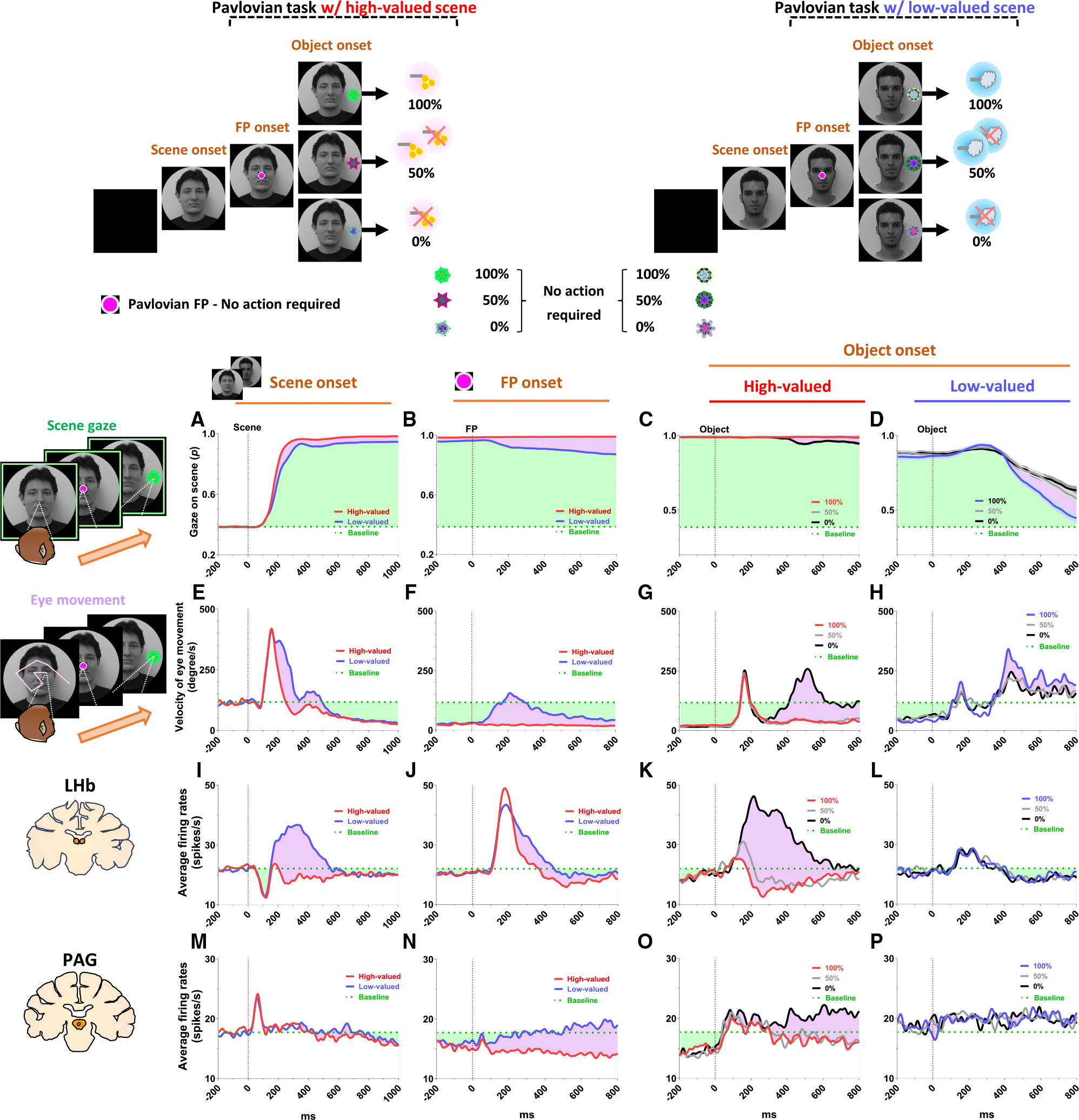
Tonic excitation of PAG signals complete extinction of reward expectancy in a Pavlovian task (A–D) The probabilities of gaze fixation on scene images during the Pavlovian task. The purple-shaded areas represent differences in gaze responses between high-valued and low-valued scenes. The green dotted line indicates baseline levels measured for 200 ms before scene onset, while the green-shaded areas represent changes in gaze responses relative to baseline during the task procedures. (E–H) Same as (A)–(D) but for the velocity of eye movement. (I–L) The LHb activity shown in Figures 2C and 2D aligned with the gaze response presented in (A)–(H). (M–P) The PAG activity corresponds to the data presented in (I)–(L).

The phasic LHb response was advantageous for rapidly encoding value information from the scene images, with a short latency of around 150 ms (Figure 1). At the beginning of the task procedures, following the appearance of the scene image, the neuronal activity of the LHb decreased by approximately 35% in both high-valued and low-valued scenes around 100 ms. Subsequently, from about 150 ms after scene onset, LHb neurons showed distinct responses depending on the reward value of the scene. The activity increased by up to 67% for low-valued scenes (Figure 1I, 300 ms), whereas it was suppressed by up to 20% for high-valued scenes (Figure 1I, 250 ms). The difference between these two scenes persisted for about 450 ms, after which their firing rates quickly recovered to baseline levels of neuronal activity, similar to those before the onset of the scene. This enabled the LHb to cease its response to the scene images before the next stimulus appeared and to prepare for subsequent responses in the following steps (Figures 4J and 5J, before 0 ms).

In contrast, unlike the LHb, PAG neurons did not respond phasically to the reward value of the scene images. After scene onset, around 50 ms, neuronal activity increased consistently by approximately 26% for both high- and low-valued scenes, and this response quickly returned to baseline. Subsequently, around 300 ms, activity was slightly inhibited by high-valued scenes and slightly excited by low-valued scenes, although the difference between the two conditions was not significant (Figure 1J). PAG responses also showed a stronger tendency to maintain tonic suppression in both high- and low-valued scenes until the next visual stimulus appeared (Figures 4N and 5N, before 0 ms). This result implies that PAG activity could tonically regulate the persistence of the task-engaged state in animals from a reward-informative stimulus to a subsequent reward-informative stimulus.

### Tonic PAG activity passes over prediction error and signals the continuity of reward expectancy until the goal is ultimately obtained

We found that the PAG response is specifically tuned to signal the continuity of reward expectancy, even when negative reward prediction error occurs during the sequential procedures of reward-seeking behaviors. As described above, the PAG neurons exhibited tonic suppression despite the negative reward prediction error induced by the low-valued scene (Figure 1J). Especially through the instrumental task, we observed additional evidence showing that PAG activity was able to maintain tonic suppression despite further negative reward prediction errors occurring, until the reward was ultimately obtained (Figures 2A, 2B, and 4M–4P).

The instrumental task was designed to observe behavioral and neural responses when a temporary negative reward prediction error occurs during sequential task procedures, but the possibility of obtaining a reward outcome is 100% guaranteed. The instrumental task began with the appearance of a square-shaped FP on the scene images. Once the monkeys completed gaze fixation on the FP, it disappeared, and good or bad objects appeared randomly on the left or right side (Figures 2A and 2B, object onset). The appearance of the good object allowed the monkeys to receive a reward immediately upon fixing their gaze on it (Video S1). In contrast, the bad object required avoiding turning their gaze toward it (Video S2). The monkeys could avoid the bad object by either not making a saccade toward it for 1 s or making a saccade but breaking the fixation within 500 ms. After the monkeys successfully avoided gazing at the bad object, the FP reappeared. Subsequently, the monkeys were finally able to find the good object and received a reward by fixating on it. Even if they failed, the same trial was repeated until they succeeded, ultimately receiving a reward outcome with a 100% probability. Consistent with previous studies,^47^ we observed that in high-valued scenes, where a larger reward was predicted, monkeys exhibited a higher rate of correct operant responses (Figure S4A) and made saccades to the good object with faster reaction times compared to low-valued scenes, where only a small reward was predicted (Figure S4B). Nevertheless, we confirmed that they exhibited over 90% correct operant responses in both high-valued and low-valued scenes, regardless of whether larger or smaller rewards were predicted (Figure S4A), as the monkeys performed the task with a 100% reward probability on each trial.

As a result, we found that the phasic response of the LHb effectively encoded the value difference between good and bad objects (Figures 4K and 4L). The appearance of a bad object represented the balance between costs and benefits, as revealed in reward schedule tasks.^48^ As obtaining the same reward required a greater cost compared to when a good object appeared immediately, the appearance of a bad object results in additional temporal and effort costs. Consistent with previous studies, in the high-valued scenes, we observed that the reaction time for a subsequent good object was significantly increased following the appearance of a bad object (Figure S4C).

On the other hand, the tonic activity of the PAG remained suppressed throughout the sequential procedures of the instrumental task, even when good or bad objects appeared, ultimately leading to a 100% probability of obtaining a reward outcome (Figures 4O and 4P). We observed that the tonic suppression of the PAG, which began with the initial scene onset (Figure 4M), was sustained until the FP onset (Figure 4N) and continued even after either the good or bad objects appeared (Figures 4O and 4P). Although PAG activities were slightly excited by the appearance of either good or bad objects, their activities quickly declined and returned to a level lower than baseline (Figures 4O and 4P, before 0 ms). As a result, the firing rates of the PAG neurons remained lower than baseline levels when the FP reappeared following the avoidance of the bad object.

### Distinct LHb and PAG responses signal disappointment and continuity of remaining reward expectancy

On the contrary, we also confirmed that PAG activity becomes tonically excited when the reward expectancy is completely extinguished during an ongoing motivated behavior and remains in a tonically excited state until the end of the trial. This was evident in the Pavlovian task, which dramatically altered the remaining reward expectancy, sustaining it in high-valued scenes and completely extinguishing it in low-valued scenes.

In the high-valued scenes, the Pavlovian task resulted in different probabilities of obtaining rewards (100%, 50%, and 0%) depending on the object presented (Figure 2C; Video S3). Ultimately, the appearance of the FP indicating the Pavlovian task in the high-valued scenes resulted in an average probability of obtaining probability of 50% (the average of 100%, 50%, and 0%). This implies that it caused disappointment compared to the 100% probability of obtaining rewards in the instrumental task (Figures 3F–3H and 3N–3P), but there was still a possibility of obtaining rewards.

On the other hand, in the low-valued scene, when the Pavlovian task began with the appearance of the FP, the probability of obtaining a reward was completely extinguished to 0%, leaving only the prediction of punishment (Figure 2D; Video S4). This not only caused disappointment compared to the 100% probability of obtaining rewards in the instrumental task but also implied that the possibility of obtaining rewards within the trial was completely extinguished (Figures 3N–3P).

**Figure 6.**
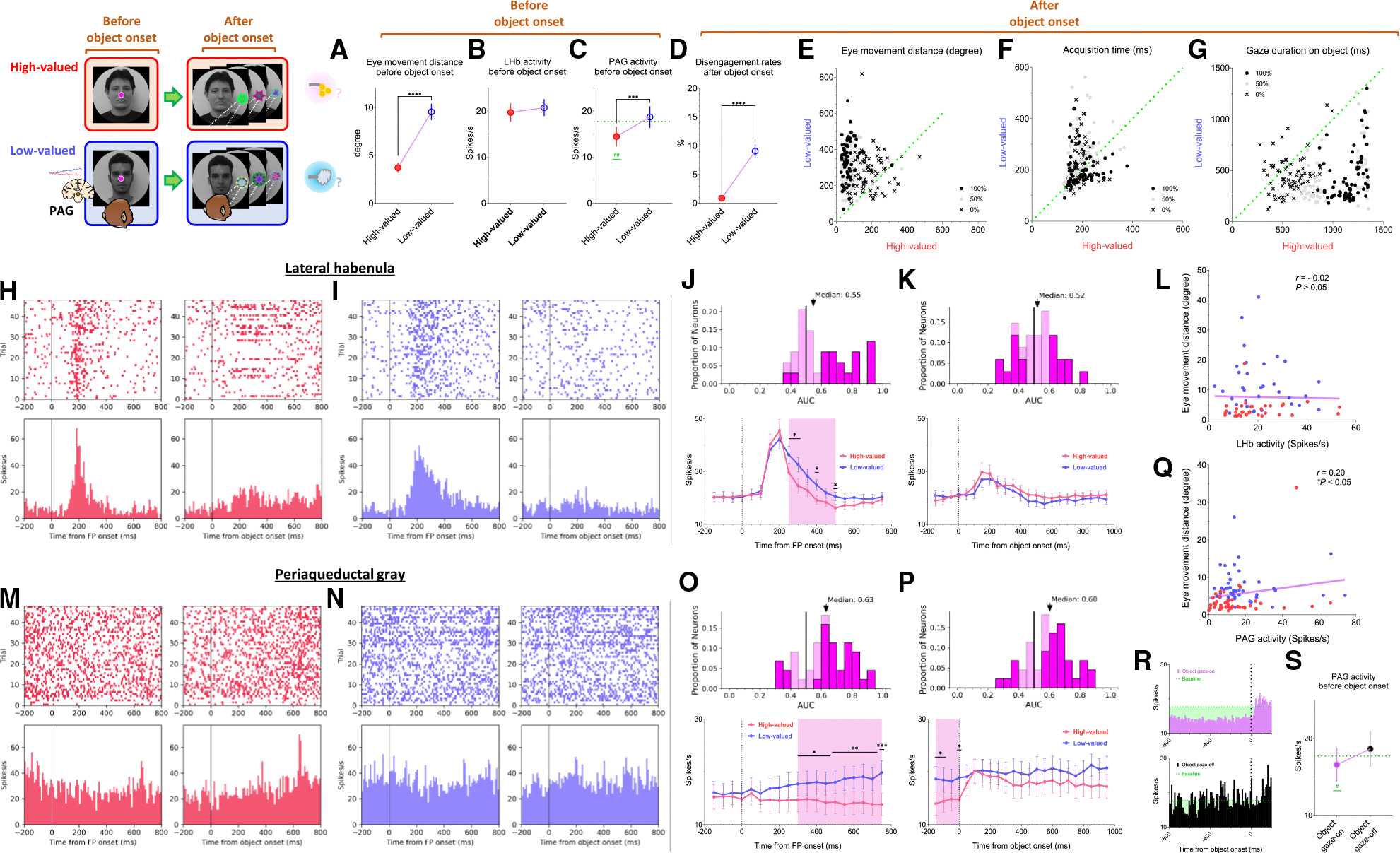
Implications of tonic PAG activity on subsequent behaviors through the inhibitory control of eye movements (A) The average eye movement distance before object onset in the Pavlovian task. The distances were quantified during the −200 to 0 ms period preceding object onset (high valued, 3.708 ± 0.5140; low-valued, 9.532 ± 0.8520; Wilcoxon matched-pairs signed rank test; ****p < 0.0001, *n* = 72). (B and C) The average firing rates of LHb and PAG neurons before object onset during the Pavlovian task. The firing rates were quantified for PAG neurons, as shown in Figures 5K and 5L, during the −200 to 0 ms period preceding object onset (LHb: high valued, 19.66 ± 2.000; low valued, 20.71 ± 1.834, Wilcoxon matched-pairs signed rank test; *p* > 0.05, *n* = 34; and PAG: high valued, 14.43 ± 2.154; low valued, 18.67 ± 2.338; ***p < 0.001, *n* = 44; one-sample Wilcoxon tests vs. PAG baseline firing rates, 17.71 spikes/s; ^##^*p* < 0.01). (D) Disengagement rate for objects in high- and low-valued scenes. The disengagement rate was calculated as the percentage of trials in which monkeys did not fixate on the object during the 1.5 s that the object was on the screen. Monkeys exhibited higher disengagement rates in low-valued scenes, indicating reduced task engagement (LHb: high valued, 0.8681 ± 0.2322; low valued, 9.057 ± 1.177, Wilcoxon matched-pairs signed rank test; ****p < 0.0001, *n* = 72). (E) The average eye movement distance after object onset in the Pavlovian task. The distances were quantified for 1 s after object onset, as shown in Figures 5G and 5H (*n* = 72). (F) The time to initiate gaze on the object after object onset in the Pavlovian task. The start time for the monkeys’ gaze reaching the object was analyzed (*n* = 72). (G) The duration of gaze fixation on the objects after object onset in the Pavlovian task. The gaze-holding duration was measured for 1.5 s (*n* = 72). (H and I) Raster plots and corresponding histograms of an example LHb neuron, aligned to FP onset and object onset, with a 10 ms bin width. </p/> (J and K) Top, area under the ROC curve (AUC) distributions. Bottom, average firing rates of LHb and neurons calculated with a 50 ms bin width. Statistical differences between groups were analyzed using repeated-measures two-way ANOVA (FP onset: main effect of group: F(1.000, 33.00) = 5.533, *p* = 0.0248; main effect of time: F(1.954, 64.47) = 32.98, *p* < 0.0001; interaction: F(3.884, 128.2) = 6.449, *p* = 0.0001; and object onset: main effect of group: F(1.000, 33.00) = 2.906, *p* = 0.0977; main effect of time: F(3.032, 100.1) = 7.278, *p* = 0.0002; interaction: F(5.500, 181.5) = 1.221, *p* = 0.2993, *n* = 34), followed by Holm-Šídák’s multiple comparisons test (*p < 0.05). Dark shading in the AUC distributions indicates the proportion of neurons showing significant differences between conditions. (L) The correlation between the average firing rates of LHb neurons (from B) and the average eye movement distances (from A) (Pearson correlation analysis; *r* = −0.02, *p* > 0.05, *n* = 34). (M and N) Same as (H) and (I) but for PAG neurons. (O and P) Same as (J) and (K) but for PAG neurons. Statistical differences between groups were analyzed using repeated-measures two-way ANOVA (FP onset: main effect of group: F (1.000, 43.00) = 17.71, *p* = 0.0001; main effect of time: F(3.014, 129.6) = 0.9304, *p* = 0.4284; interaction: F(4.301, 184.9) = 6.468, *p* < 0.0001; and object onset: main effect of group: F(1.000, 43.00) = 6.490, *p* = 0.0145; main effect of time: F(3.768, 162.0) = 3.538, *p* = 0.0098; interaction: F(5.543, 238.4) = 2.219, *p* = 0.0468, *n* = 44), followed by Holm-Šídák’s multiple comparisons test (*p < 0.05). Dark shading in the AUC distributions indicates the proportion of neurons showing significant differences between conditions. (Q) The correlation between the average firing rates of PAG neurons (from C) and the average eye movement distances (from A) (Pearson correlation analysis; *r* = 0.20, *p < 0.05, *n* = 44). (R) Histogram of PAG neuronal activity before object onset (10 ms bins). Top (object gaze on), when monkeys made saccades and fixated on the object, reflecting an engaged state. Bottom (object gaze off), when monkeys did not fixate on the object, reflecting a disengaged state. (S) PAG neuronal activity during the 500 ms preceding object onset. In the engaged state, when monkeys fixated on the object, activity was significantly reduced relative to baseline (object gaze on, 16.58 ± 2.179, *n* = 44). Statistical significance was assessed using one-sample Wilcoxon tests against PAG baseline firing rates (17.71 spikes/s, ^#^*p* < 0.05). In the disengaged state, when monkeys did not fixate on the object, activity was not significantly different from baseline (object gaze off, 18.64 ± 2.310, *n* = 36). Object gaze off was observed in 36 of 44 PAG recording sessions. Data are presented as the mean ± SEM.

As a result of the common disappointment in both high- and low-valued scenes, LHb activities were excited by the FP indicating the Pavlovian task (Figures 5J and 6H–6L). However, PAG exhibited different patterns of excitation and inhibition in response to the FP, depending on whether the Pavlovian task was initiated in high-valued or low-valued scenes (Figures 5N and 6M–6O).

In the low-valued scenes, the PAG became excited when the expectancy of reward outcomes was completely extinguished by the appearance of the FP indicating the Pavlovian task (Figure 5N). Then, this tonically excited state of PAG activity was sustained until the end of the trial, remaining slightly above baseline levels when an object appeared after the FP (Figure 5P, before 0 ms). The PAG activity was not responsive to variations in punishment probability (100%, 50%, or 0% airpuff), differentiated by the appearance of objects (Figure 5P), but had no significant impact on the previously extinguished remaining reward expectancy.

In contrast, in the high-valued scenes, the Pavlovian task induced disappointment but still allowed the monkeys to maintain the expectancy of obtaining rewards, even though the probability was reduced. As a result, when the FP indicating the Pavlovian task appeared in the high-valued scenes, the PAG remained in a suppressed state until an object appeared (Figure 5O, before 0 ms). Subsequently, the neuronal activities of the PAG were significantly differentiated by the 100%, 50%, and 0% reward objects. When the objects associated with 100% or 50% reward probabilities appeared, the PAG activity remained in a tonically suppressed state, but it became excited in response to the 0% reward object, which also signifies the complete extinction of reward expectancy within that trial (Figure 5O).

Consequently, we suggest that the distinct phasic LHb and tonic PAG responses could play separate roles in signaling disappointment and remaining reward expectancy. How, then, do changes in the tonic activity of the PAG contribute to behavioral responses when animals encounter an event that switches the remaining reward expectancy on or off?

### Tonic PAG activity facilitates subsequent actions based on inhibitory motor control

Finally, we propose that the tonic activity of PAG neurons could signal the continuity of remaining reward expectancy and facilitate subsequent actions based on inhibitory motor control. Our findings have so far revealed that PAG neurons exhibited tonic inhibition during the instrumental task, which guaranteed a 100% probability of obtaining a reward (Figures 4M–P). This tonic inhibition of the PAG corresponded to the continuity of reward expectancy, facilitating monkeys’ persistent visual attention throughout the task trials (Figures 4A–4D). Additionally, during this period, we observed that their overall eye movements were suppressed (Figures 4E–4H) while the PAG exhibited tonic inhibition. The distance of eye movements was calculated based on changes in eye position recorded in milliseconds. The changes in eye position are depicted by yellow dots in Videos S1, S2, S3, and S4. This inhibitory control of eye movements could reflect an increase in gaze fixation on the scene images, indicating the persistence of visual attention. Along with this, our further evidence suggests that the inhibitory control of eye movements could play additional crucial roles in facilitating the execution of subsequent actions.

This was evident in the Pavlovian task, which did not require any instrumental actions and allowed us to observe the natural behaviors of the monkeys. When the FP indicating Pavlovian task appeared in low-valued scenes, the monkeys’ remaining reward expectancy was completely extinguished. As a result, PAG activity was tonically excited (Figures 5N and 5P), and the monkeys exhibited an increase in eye movements (Figures 5F and 5H), which in turn resulted in a decrease in scene gaze compared to high-valued scenes (Figures 5B and 5D). Consequently, PAG activity (Figures 5O, 5P, and 6P, before 0 ms) and eye movements (Figures 5G and 5H, before 0 ms) at the time of object onset were greater in low-valued scenes than in high-valued scenes (Figure 6A). We also found that monkeys exhibited reduced engagement when objects were presented in low-valued scenes, as indicated by a lower rate of gazing at the objects for 1.5 s while they were on the screen (Figure 6D). Ultimately, we found that PAG activity observed at the time of object onset was significantly correlated with the distance of the monkeys’ eye movements before the object onset (Figure 6Q), whereas LHb activity did not show a significant correlation with eye movement changes (Figure 6L). We further confirmed that, when comparing residual PAG activity after statistically regressing out eye movement distance, the previously significant difference in PAG activity between high- and low-valued scenes (Figure 6C) was weakened and no longer statistically significant (Figure S5B). In addition, PAG activity was significantly correlated with eye movement distance in high-valued scenes (Figure S5E). In contrast, this correlation was not significant in low-valued scenes, where reward expectancy was completely extinguished (Figure S5F). These findings suggest that PAG activity does not directly encode the continuity of remaining reward expectancy but rather reflects the inhibitory control of eye movements coordinated with reward expectancy. To further support the relationship between inhibitory firing patterns in PAG neurons and task engagement, we analyzed PAG activity during the 500 ms preceding object onset (Figure 6R). When monkeys made saccades and fixated on the object, reflecting a task-engaged state, PAG activity was significantly lower than baseline (Figure 6S). In contrast, when monkeys were disengaged and did not fixate on the object, PAG activity was not significantly reduced relative to baseline (Figure 6S).

Even after the object appeared, the differences in eye movements between the high-valued and low-valued scenes persisted and were sustained until the end of the trial (Figure 6E). Alongside these differences in their task-engaged state, when the object subsequently appeared in the Pavlovian task, the monkeys in the low-valued scenes experienced slowness in locating their gaze on the object (Figure 6F). Furthermore, after making a saccade, their gaze did not remain fixated as long as it did in the high-valued scenes (Figure 6G).

## DISCUSSION

### Tonic PAG activity and remaining reward expectancy in overcoming disappointment

This study proposes that when animals encounter events that induce negative reward prediction error, they could overcome negative prediction errors and continue reward-seeking behaviors through a balance between distinct phasic and tonic signals from the LHb and PAG.

The LHb is well known as one of the primary inputs to the substantia nigra pars compacta (SNc) and ventral tegmental area (VTA) dopamine neurons for signaling negative reward prediction error.^19,49–52^ This neuronal pathway is essential for adaptability, flexibility, and learning of animal behaviors, as it can modulate neuronal plasticity in the basal ganglia via dopamine transmission.^53–56^ The LHb responds to events that induce negative reward prediction error with phasic firings based on the discrepancy between actual outcomes and predicted values.^3,25,57,58^ Consequently, when an animal obtains a reward larger than expected, it suppresses LHb activity, enhances dopamine release to the striatum, and activates the direct pathway within the basal ganglia system. This pathway projects to the superior colliculus (SC), thalamus, and pedunculopontine nucleus (PPN), thereby facilitating the associated actions via the substantia nigra pars reticulata (SNr) and the internal segment of the globus pallidus (GPi).^59–61^ Conversely, when the reward is smaller than expected, LHb activity is stimulated, leading to reduced dopamine release and activation of the indirect pathway, which includes the external segment of the GP (GPe) and the subthalamic nucleus (STN), thereby suppressing the associated actions.^62–66^

However, to optimize reward acquisition, animals often need to overcome negative reward prediction error and sustain reward-seeking behaviors by considering additional factors (e.g., good or bad) beyond these prediction errors (e.g., better or worse). For instance, during reward-seeking behaviors, animals may encounter an event that is relatively worse than expected but could still hold an absolute value worth pursuing.^67^

The present study reveals that tonic activity in the PAG is proficient in signaling continuity of remaining reward expectancy throughout task trials, persisting until the desired reward is ultimately obtained (Figures 4M–4P and 6M–6P). We propose that the PAG plays a key role in regulating the task-engaged state, enabling animals to remain consistently engaged in a sequence of task procedures with heightened visual attention, even in the face of transient events that induce negative reward prediction errors (Figures 4A–4D and 5A–5D). In the instrumental task, the tonic activity of PAG neurons remains in a suppressed state under varying effort costs to obtain the same reward, reflecting their role in regulating effortful behavior. Previous research has defined behaviors requiring variable costs to achieve the same reward as “effort,” a mechanism corresponding to the animal’s ability to overcome difficulties.^48,68^ These innovative studies have further identified key brain structures, including the caudate,^69^ ventral pallidum,^70^ locus coeruleus,^71–73^ and centromedian thalamus,^47^ as critical contributors to orchestrating effortful actions.

**Figure 7.**
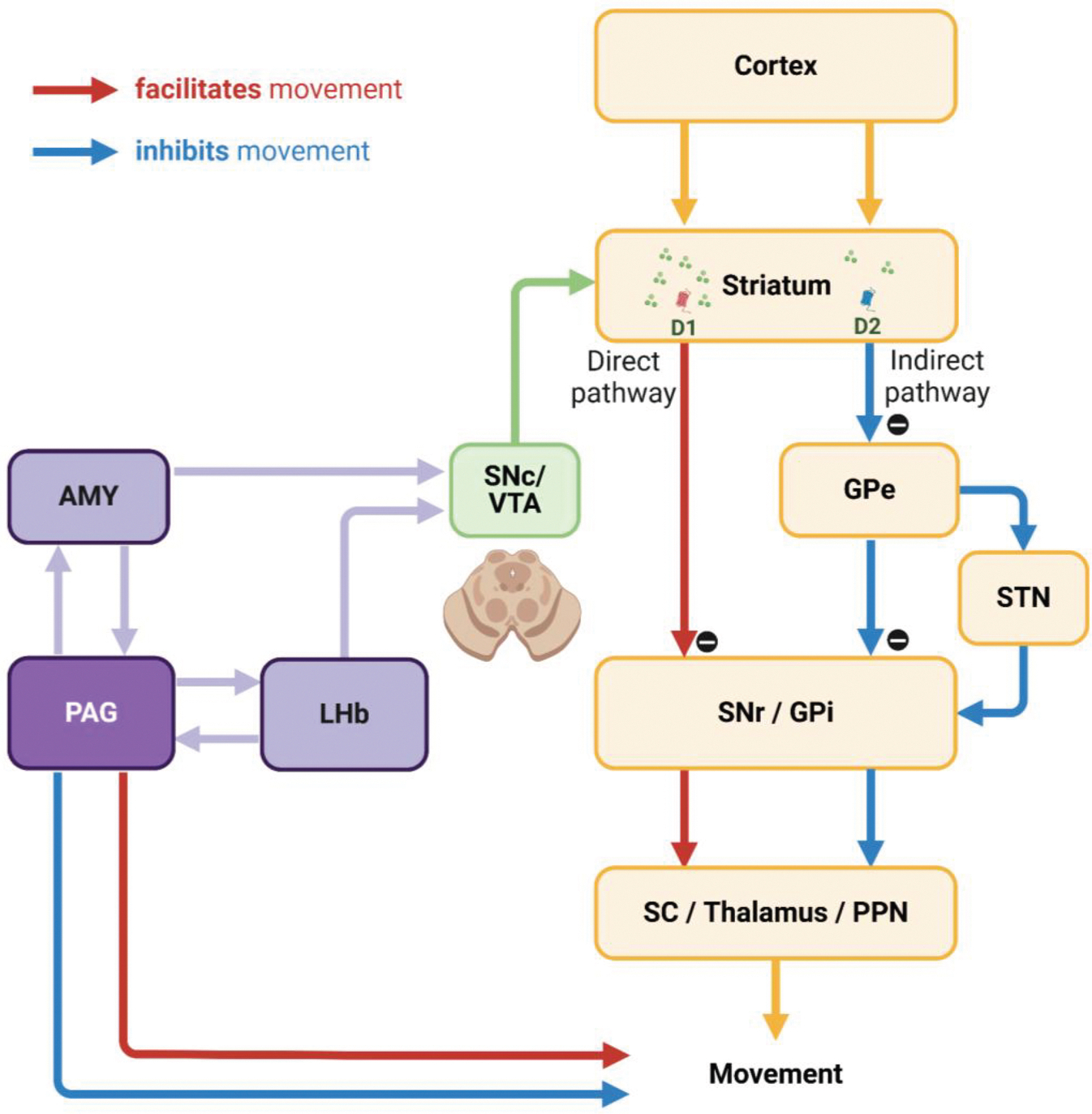
Hypothetical neuronal mechanisms underlying inhibitory control of movements The interconnection between the PAG and AMY could play a key role in the inhibitory control of movements via dopamine neurons in the SNc and VTA. This pathway could enable dopamine neurons to integrate tonic signals from the PAG with phasic signals from the LHb. Furthermore, the PAG could be essential for integrating emotional information from the AMY and LHb, thereby orchestrating movement control through additional interactions with brain regions such as the forebrain, cerebellum, and other brainstem areas. This figure was created with BioRender.com.

On the one hand, the PAG has reciprocal connections with the LHb, which should not be overlooked as a potential node for exchanging tonic signals between these brain regions, particularly within the cortico-basal ganglia-thalamus loop, and for guiding animals to overcome events that induce negative reward prediction errors.^74–78^ On the other hand, the PAG also establishes slow dynamics in neuronal networks with other structures, in which such sustained activity is observed, including the dorsal raphe nucleus,^79,80^ ventral pallidum,^81,82^ and amygdala (AMY).^83–86^ In previous studies, we specifically reported similar tonic activity patterns in AMY neurons that were associated with facilitated, extraordinary goal-directed eye movements within the range of express saccades.^87,88^ Therefore, we suggest that this tonic signaling from the AMY could be a strong candidate for modulating dopamine activity via the amygdalonigral pathway,^89,90^ and dopamine neurons could, in turn, integrate tonic signals from the PAG and phasic signals from the LHb (Figure 7). Finally, further studies on the reciprocal connections of the PAG with the LHb and AMY could deepen our understanding of how animals overcome negative reward prediction errors and sustain reward-seeking behaviors, ultimately achieving desired outcomes. Indeed, the PAG has recently been highlighted as a potential therapeutic target for major depressive disorder,^91–93^ which could expand treatment approaches and complement LHb studies aimed at improving sustained antidepressant effects.^94–96^

Lastly, among brain regions related to the neural basis of effort, the locus coeruleus is particularly well known for regulating autonomic responses critical for animal survival and for influencing not only reward, motivation, and decision-making but also arousal and attention. Similarly, the PAG, as a key region connecting the brain and spinal cord, plays an important role in autonomic responses and arousal, suggesting that PAG mechanisms in arousal may be closely interconnected with the regulation of animals’ task engagement.^97^ From this perspective, we further tested four groups in our experiment by manipulating the presence or absence of airpuff punishment within each high-valued and low-valued scene: high-value reward without punishment (Rwd++ Pun−), high-value reward with punishment (Rwd++ Pun+), low-value reward without punishment (Rwd+ Pun−), and low-value reward with punishment (Rwd+ Pun+). However, our results showed that, at scene onset, PAG neurons did not exhibit significant differences across groups in response to punishment, whereas differences related to value were more pronounced and accompanied by behavioral changes (Figures S1 and S2). Nevertheless, despite these findings, PAG neurons in the Pavlovian task displayed tonic excitation in low-valued scenes containing punishment-associated objects, even as reward expectancy diminished, in contrast to high-valued scenes. This observation suggests that arousal-related effects may still play a critical role in this mechanism. Future studies should directly address how such arousal-related influences contribute to tonic signaling in PAG neurons.

### Tonic suppression of PAG activity in bridging sequential reward-seeking behaviors

Next, our findings suggest that the tonic activity of the PAG could play a crucial role in bridging sequential motor actions involved in reinforcement learning. This is evident in the correlation between the tonic signaling of the PAG and the anticipatory gaze of the monkeys, which consistently maintained their gaze on the background scene images where their tasks took place (Figures 1K–1N). Thereby, the anticipatory gaze enabled the monkeys to prepare for subsequent actions, allowing them to detect the FP with greater speed and gaze rates (Figure 3).

This is an essential component in the motor skill behaviors of animals, as it allows a sequence of actions to be interconnected and executed as cohesive behavioral units (i.e., motor chunk).^98–100^ As a result, animals could improve the speed and accuracy of a series of sequential procedures in reward-seeking behaviors.^101,102^ This is exemplified by our previous study, which examined motor skill acquisition in monkeys using a 2 × 5 task.^103^ The 2 × 5 task involved training the monkeys to learn and remember the sequence of pressing five pairs of illuminated buttons among a total of 16 buttons in a predetermined order. Following a series of training sessions, the monkeys exhibited skilled behavior in which, upon pressing one button, they were able to turn their gaze to the next button they would press before it illuminated. As a result, they could perform subsequent actions with exceptional speed and accuracy, leading to faster reward acquisition.

Furthermore, this reinforcement between behavioral units could also play a pivotal role in gating subsequent behaviors sequentially. For example, monkeys initiate eye contact before engaging in social behaviors such as lip smacking.^104^ Sequentially, lip smacking induces synchronization of the animal’s behavior, facilitating emotional exchange and cooperative actions, which, in turn, promote a series of various subsequent behaviors.^105–107^ Thus, the tonic signaling of the PAG, which supported the monkeys’ anticipatory gaze toward face scene images (Figure 1), could play a crucial role in enhancing the likelihood of animals engaging in subsequent social behaviors. These motor skills, including social behaviors, are important sources of natural rewards and pleasure in the lives of humans and animals.^108–110^ In this way, social interactions between animals have been shown to improve stress coping,^111–113^ mitigate aversive responses to unpleasant stimuli, and enhance the persistence of ongoing motivated behaviors.^114–118^ Therefore, from the perspective of reinforcement learning, we suggest that the tonic activity of the PAG can provide additional reward resources for overcoming events that induce negative reward prediction error and continuing ongoing motivated behaviors by facilitating the connection between sequential motor actions.

### Tonic PAG activity in inhibitory control of unnecessary movements

Lastly, we suggest that tonic PAG activity can contribute to the inhibitory control of unnecessary movements, which could potentially implicate its significant role in movement disorders. In the present study, we observed that monkeys exhibited strong inhibition of unnecessary eye movements during highly motivated anticipatory scene viewing (Figures 4E–4H and 6E–6G). The inhibitory motor control can play a crucial role in regulating impulsivity, guiding action selection, and prioritizing the sequence of actions in human and animal behavior.^119–121^ Thereby, this process ultimately improves the speed and accuracy of desired behaviors and smooths out the motion of humans and animals in response to the overwhelming influence of information processed through various sensory inputs that flow into the brain.^122–125^

These findings could further provide valuable insights into the implications of PAG activity in movement disorders, such as Parkinson’s disease. Parkinson’s disease is characterized by abnormalities in the initiation and speed of actions (e.g., akinesia and bradykinesia).^54^ On the other hand, another significant symptom observed in this disease is impairment in suppressing unnecessary movements, such as tremors and dyskinesia. These symptoms manifest abnormalities not only in limb movements but also in eye movements.^126–128^ For example, a patient with Parkinson’s disease in a previous study exhibited frequent abnormal repetitive saccades and fixation patterns during word reading, leading to more frequent unnecessary eye movements compared to a healthy individual.^129^ As a result, this impaired their overall reading speed and comprehension.

A key characteristic of Parkinson’s disease is the degeneration of dopaminergic neurons in the SNc. Additionally, the PAG, along with surrounding brainstem regions, has also been reported as an area showing significant changes in patients and animal models with Parkinsonian symptoms.^36,41,130–134^ However, there is still a lack of research on the role of the PAG in Parkinsonian symptoms. The dopamine deficits in patients with Parkinson’s disease have primarily been reported in the caudal-dorsal-lateral part of the SNc, which projects to the tail of the striatum (i.e., the caudal part of the striatum).^135–138^ This neuronal pathway from the caudal-dorsal-lateral part of SNc to the tail of the striatum plays a crucial role in regulating automatic movements.^139–143^ Consequently, neurodegeneration in this pathway can impair the function of the indirect pathway, which is responsible for the inhibitory control of unnecessary automatic movements,^14,144,145^ and the decrease in PAG activity may affect its interaction with these basal ganglia circuits.

Importantly, the PAG is also recognized as a critical integrator of emotional responses and outputs in the emotional motor system, projecting to the spinal cord, forebrain, cerebellum, and other brainstem regions to control movement (Figure 7).^146–149^ For instance, dysfunction of the PAG might impair eye movement control in Parkinsonism through its connections, distinct from basal ganglia output, to the brainstem areas associated with the oculomotor system.^150–152^ Future studies investigating the specific mechanisms of PAG activity and its interplay with basal ganglia circuits could significantly enhance our understanding of the pathophysiology of movement disorders and identify new therapeutic targets.

### Limitations of the study

While our study rigorously elucidated the distinct phasic and tonic signaling of neurons in the LHb and PAG and their functional characteristics, several limitations should be noted. First, recordings were limited to the LHb and PAG, and interactions with broader neuronal networks remain unexplored. Second, although our task was conducted in various naturalistic environments, additional ecological factors need to be studied further to better understand how diverse goal-directed behaviors, motor skills, and decision-making processes are orchestrated in the real world.^153^ Third, while correlations between neuronal activity and behavior were statistically demonstrated, future studies employing interventions from cell-specific to circuit-specific levels will be necessary to establish the underlying neural mechanisms and to explore potential therapeutic benefits for neurological and psychiatric disorders.^61,154,155^ Lastly, the present study suggests the functional characteristics of LHb and PAG neurons in neurocognitive functions and emotion and indicates their potential relevance to human diseases, such as Parkinson’s disease. To bridge the gap between the clinical and psychological aspects observed in the present study, multidisciplinary cross-species approaches and investigations will be key in future studies.

## RESOURCE AVAILABILITY

### Lead contact

Requests for further information and resources should be directed to and will be fulfilled by the lead contact, Hyunchan Lee (hyunchan.lee@georgetown.edu).

### Materials availability

This study did not generate new, unique reagents.

### Data and code availability

- All animal data have been deposited at Zenodo at https://doi.org/10.5281/zenodo.17944350 and are publicly available as of the date of publication.
- All original code has been deposited at Zenodo and is publicly available at https://doi.org/10.5281/zenodo.17944350 as of the date of publication.
- Any additional information required to reanalyze the data reported in this paper is available from the lead contact upon request.

## STAR★METHODS

### EXPERIMENTAL MODEL AND STUDY PARTICIPANT DETAILS

Two male Macaca mulatta monkeys were used for this study. (CH, 10-year-old, 15 kg and KI, 10-year-old, 9.5 kg). All animal care and experimental procedures were approved by the Animal Care and Use Committee of the National Eye Institute and complied with the Public Health Service Policy on the Humane Care and Use of Laboratory Animals. Apple juice (high-value, 600 μL; low-value, 200 μL) and airpuff (10–20 psi, 100 ms) were used as reward and punishment outcomes in the task. Partial data from the LHb and behavioral recordings were used in related publications that tested the same task protocol.^25,106^ The monkeys’ eye positions were recorded across 72 sessions using an EyeLink 1000 Plus eye tracker (SR Research) and were simultaneously recorded with neuronal signals via Blip software (www.robilis.com/blip/).^59^ The influence of sex could not be assessed due to the limited number of subjects.

### METHOD DETAILS

#### Scene-based instrumental/Pavlovian task

A trial of the scene-based instrumental/Pavlovian task began with the appearance of a scene image (size: 40°; (2 faces +2 landscapes) × 4 scene groups = 16 scene images per block), which was maintained as the background scene throughout the trial. After 1 s of free viewing, an FP indicating either the instrumental or Pavlovian task appeared at the center of the scene, and the respective task began. Each block of the task consisted of 384 trials, with tasks presented in a pseudo-random order (instrumental task, 192 trials; Pavlovian task, 192 trials). Additionally, 32 non-cued free outcomes (8 high-value rewards, 8 low-value rewards, 16 punishments) were randomly delivered between task trials without any visual stimuli.

#### Background scene images

We created four groups of background scene images, each contextually associated with different reward and punishment outcome experiences (Figures S1 and S2): high-value reward without punishment (Rwd++ Pun−), high-value reward with punishment (Rwd++ Pun+), low-value reward without punishment (Rwd+ Pun−), and low-value reward with punishment (Rwd+ Pun+). For each of these four contexts, four scene images were used (two face scenes and two landscape scenes), resulting in a total of 16 scene images. Each scene image was associated with a unique set of objects. In the instrumental task, each scene had one good object and one bad object, while in the Pavlovian task, each scene had three objects associated with 100%, 50%, and 0% reward or punishment probabilities. Thus, each of the 16 scenes had a total of five associated objects, amounting to 80 objects in total that were presented across 384 trials in one block (Figure S3). We tested two sets of such stimulus in the monkeys. In one of the sets, the reward contingencies were reversed between the two monkeys so that the same scenes were associated with opposite outcomes (high-valued scenes ↔ low-valued scenes). This study focused on data from high-valued scenes (Rwd++ Pun−) and low-valued scenes (Rwd+ Pun+) to address the primary research question. As a result, monkeys received a large amount of juice reward for completing instrumental tasks in the high-valued scenes and a small amount of juice reward in the low-valued scenes (Figure 1A). During Pavlovian tasks, in the high-valued scenes, monkeys received rewards of the same size as those in the instrumental tasks. In contrast, in the low-valued scenes, monkeys received airpuff punishment outcomes instead of juice rewards. The background scene images were collected from Google Earth (https://www.google.com/earth), OpenAerialMap (https://openaerialmap.org), and Face Database (https://fei.edu.br/~cet/facedatabase.html).

#### Instrumental task

The instrumental task began with the appearance of a square-shaped FP (size, 2°), requiring the monkeys to fixate on it for more than 700 ms within 1 s (Figure 4). After the fixation, one of the good or bad objects (size, 10°) appeared on either the left or right side (15°) of the background scene image. Monkeys were then required to fixate on the good object (500 ms) within 1 s to receive a reward. If the bad object appeared after fixation, monkeys had to avoid it by either not making a saccade to the object for 1 s or by fixating on the bad object for no more than 500 ms. If the monkey successfully avoided the bad object, it disappeared from the scene, and the FP reappeared at the center of the scene. The monkey was then required to fixate on the FP again. Afterward, the good object was presented again on either side of the scene, and the trial could be completed by fixating on the good object to acquire the reward. If the monkey failed to avoid the bad object or failed to fixate on the FP or the good object, the visual stimuli disappeared, accompanied by a beep sound. The trial was then repeated from the scene onset until the monkey correctly completed the task and acquired the reward. Therefore, monkeys could earn fixed amounts of reward outcomes for each scene image in the block. The good object appeared first in 1/3 of the trials and after the bad object in 2/3 of the trials. Each scene image had different fractal images^143^ for the respective good and bad objects.

#### Pavlovian task

The Pavlovian task began with the appearance of a circular FP, which required no action from the monkeys for 1 s (Figure 5). After 1 s, one of three objects (100%, 50%, or 0% probability) appeared on the left or right side of the background scene image for 1.5 s. Monkeys then received a reward or punishment outcome based on the probabilities associated with each object. One second after the outcome delivery began, the background scene disappeared, and the next trial started after approximately 7 s. Each scene image had different fractal objects representing the respective objects.

#### Electrophysiology

We recorded 34 neurons from the LHb and 44 neurons from the PAG in two monkeys: CH (LHb, 15; PAG, 30) and KI (LHb, 19; PAG, 14). Single-unit neuronal activity was recorded using glass-coated electrodes (diameter 0.38 mm, 1 MΩ, Alpha-Omega) connected to a microelectrode AC amplifier (model 1800; A-M Systems; gain, 10k; filters, 0.1 to 10 kHz) and a band-pass filter (model 3384; Krohn-Hite). The neuronal data were recorded across 72 separate sessions. Among these sessions, there were 6 sessions in which two neurons were recorded simultaneously, resulting in a total of 34 LHb neurons and 44 PAG neurons. In total, we obtained behavioral data from 72 sessions during which the monkeys’ neuronal activity was recorded. The electrode was advanced using an oil-driven micro-manipulator (MO-97A, Narishige) through a guide tube and an 8° tilted posterior chamber. Recording sites were confirmed via vertical MRI scanning (4.7 T, Bruker) with a gadolinium-filled grid (1 mm-spacing) and Elgioy deposits marking the PAG.^156^ Neuronal firing was monitored in real-time and isolated using custom voltage- and time-based windows in Blip software.

### QUANTIFICATION AND STATISTICAL ANALYSIS

We presented data as mean ± standard error of the mean. Statistical significances were analyzed using the one-sample Wilcoxon tests for single-group comparisons, the Wilcoxon matched-pairs signed rank test for paired data, repeated measures one-way analysis of variance (ANOVA) and repeated measures two-way ANOVA with Holm-Šídák’s post hoc test for comparing multiple groups and factors, and Pearson correlation analysis to evaluate correlations between variables. Analyses were performed using Prism9 (GraphPad Software), SPSS (IBM), and Python. The average firing rates of neurons and gaze probabilities were smoothed by Gaussian kernel (σ = 10 ms) using MATLAB (MathWorks). Statistical significances in one-sample Wilcoxon tests were indicated by hash symbols (^*#*^*p* < 0.05, ^*##*^*p* < 0.01), while significances for other statistical comparisons were indicated by asterisks (*p < 0.05, **p < 0.01, ***p < 0.001, ****p < 0.0001).

## Supplementary Material

1

2

3

4

5

SUPPLEMENTAL INFORMATION

Supplemental information can be found online at https://doi.org/10.1016/j.celrep.2025.116907.

## Figures and Tables

**KEY RESOURCES TABLE:** 

REAGENT or RESOURCE	SOURCE	IDENTIFIER
Deposited data		
Data	This study	Zenodo: https://doi.org/10.5281/zenodo.17944350
Code	This study	Zenodo:https://doi.org/10.5281/zenodo.17944350
Experimental models: Cell lines		
Macaque Mulatta	NIH	N/A
Software and algorithms		
BLIP	NIH, Simon Hong^59^	http://www.robilis.com/blip/
MATLAB	Mathworks	RRID:SCR_001622
Python	Python Software Foundation	https://www.python.org
GraphPad Prism	GraphPad	RRID:SCR_002798
Mango	University of Texas Health Science Center	RRID:SCR_009603
SPSS	IBM	RRID:SCR_002865
BioRender	BioRender	RRID:SCR_018361
Other		
Eyelink	SR Research	RRID: SCR_009602
Model 1800 2-Channel Microelectrode AC Amplifier	A-M Systems	https://www.a-msystems.com/p-253-model-1800-2-channel-microelectrode-ac-amplifier.aspx
Hydraulic micromanipulator (MO-973A)	Narishige	https://products.narishige-group.com/group1/MO-97A/chronic/english.html

## Highlights

- LHb and PAG neurons encode prediction error and continuity of reward expectancy
- They convey information via distinct phasic and tonic activity patterns
- Tonic PAG activity breaks through prediction errors and sustains reward expectancy
- Tonic PAG activity maintains reward expectancy until the goal is ultimately achieved
