# Supplementary material for: Lateral habenula and periaqueductal gray neurons signal reward prediction error and continuity of reward expectancy to drive reward-seeking behavior
Source: Cell Rep. Author manuscript; Available in PMC 2026 Mar 26. (PMC13019782; doi:10.1016/j.celrep.2025.116907)
Supplement: 1 [file NIHMS2151711-supplement-1.pdf]

Cell Reports, Volume *45*

Supplemental information

Lateral habenula and periaqueductal gray neurons signal reward prediction error and continuity of reward expectancy to drive reward-seeking behavior

Hyunchan Lee and Okihide Hikosaka

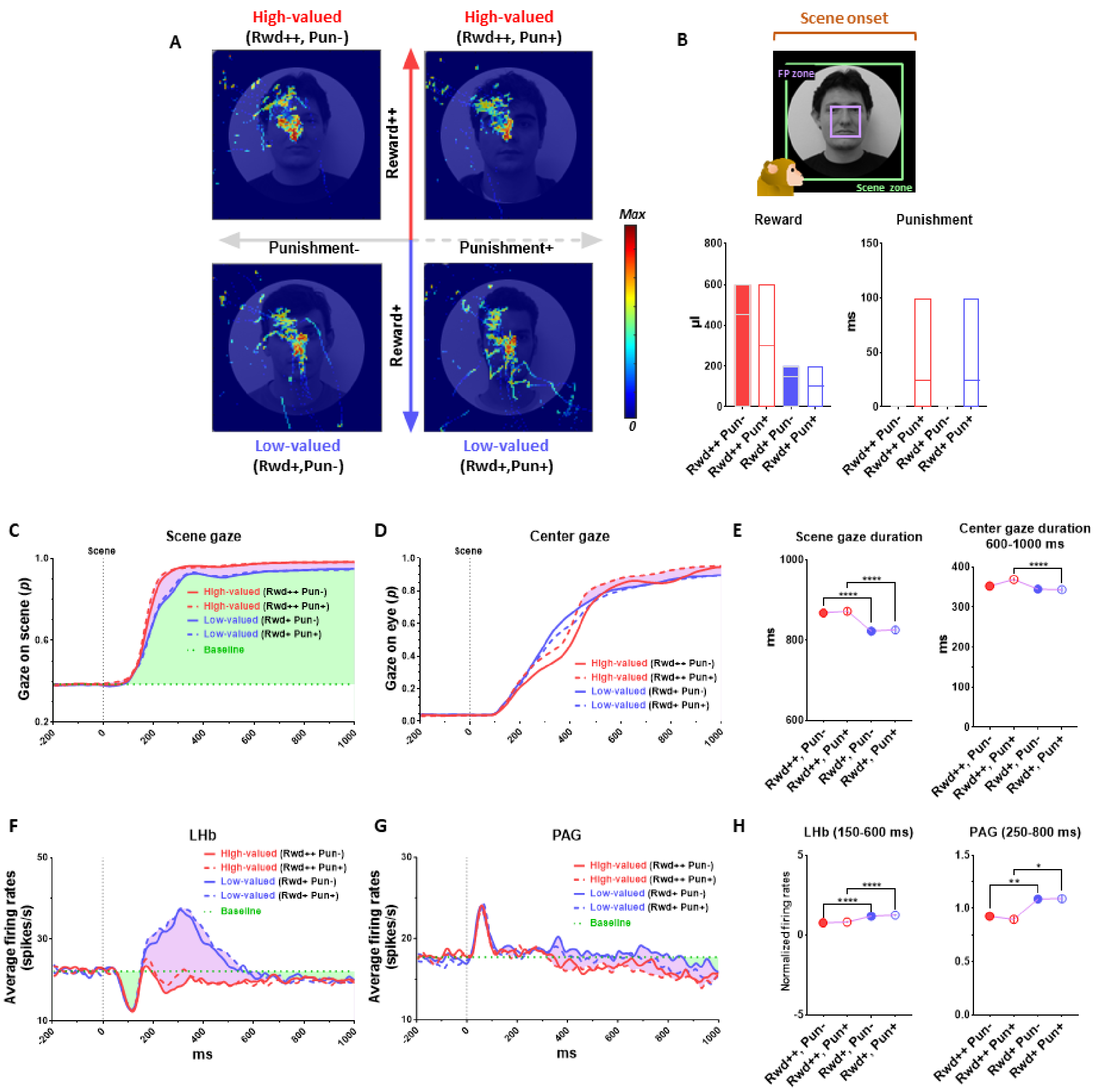

Figure S1. Neuronal responses and anticipatory gazes were predominantly regulated by expectancy of reward rather than punishment

(A) An example heatmap illustrating monkeys' gaze on face scene images. We conducted a scene-based foraging/Pavlovian task with four groups of background scene images: two high-valued scenes that provided a high-value reward, either without punishment (Rwd++ Pun-) or with punishment (Rwd++ Pun+), and two low-valued scenes that provided a low-value reward, either without punishment (Rwd+ Pun-) or with punishment (Rwd+ Pun+).

(B) The bars represent the average amount of juice reward and airpuff punishment provided for each group of scenes during the task procedures.

(C) The probabilities of gaze on face scene images during the free-viewing period. The purple shaded areas represent the differences in the responses between high-valued and low-valued scenes. The green shaded areas

represent changes in the responses relative to the baseline during the task procedures.

(D) The probabilities of gaze on the eye regions of face scene images during the free-viewing period.

(E) Left, the duration of gaze on face scene images was quantified for 1 s during the free-viewing period. Middle, the duration of gaze on the eye zone was quantified for 450 ms after scene onset. Right, the distance of eye movements was quantified for 1 s during the free-viewing period. Statistical differences between groups were analyzed using repeated measures one-way ANOVA (scene gaze duration, $F(2.001, 142.1) = 45.55$, $P < 0.0001$; center gaze duration, $F(2.129, 151.2) = 12.96$, $P < 0.0001$), followed by Holm-Šídák's multiple comparisons test ($^{****}P < 0.0001$, $n = 72$).

(F, G) The average firing rates of LHb and PAG in response to scene onset.

(H) The average firing rates of LHb and PAG neurons in response to the scene onset. The LHb response was quantified during the 150–600 ms after object onset. The PAG response was quantified during the 250–800 ms after object onset. Statistical differences between groups were analyzed using repeated measures one-way ANOVA (LHb, $F(1.235, 40.75) = 38.04$, $P < 0.0001$, $n = 34$; PAG, $F(1.722, 74.06) = 10.74$, $P = 0.0002$, $n = 44$), followed by Holm-Šídák's multiple comparisons test ($^{*}P < 0.05$, $^{**}P < 0.01$, $^{****}P < 0.0001$). Data are presented as mean ± SEM.

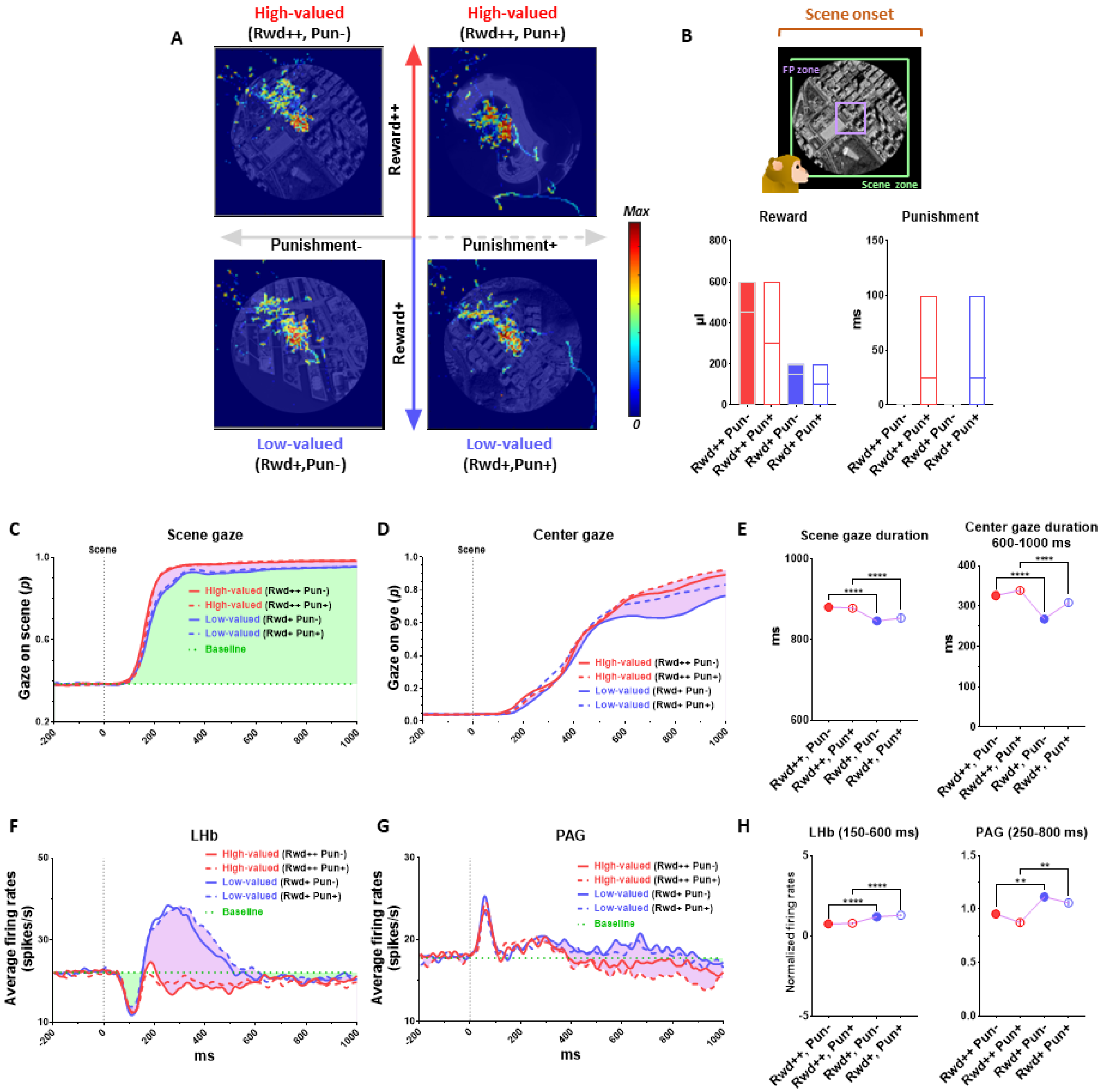

Figure S2. Neuronal responses and anticipatory gazes in landscape scene images

(A) An example heatmap illustrating monkeys' gaze on landscape scene images. We conducted a scene-based foraging/Pavlovian task with four groups of background scene images: two high-valued scenes that provided a high-value reward, either without punishment (Rwd++ Pun–) or with punishment (Rwd++ Pun+), and two low-valued scenes that provided a low-value reward, either without punishment (Rwd+ Pun–) or with punishment (Rwd+ Pun+).

(B) The bars represent the average amount of juice reward and airpuff punishment provided for each group of scenes during the task procedures.

(C) The probabilities of gaze on landscape scene images during the free-viewing period. The purple shaded areas represent the differences in the responses between high-valued and low-valued scenes. The green shaded areas represent changes in the responses relative to the baseline during the task procedures.

(D) The probabilities of gaze on the central region of landscape scene images during the free-viewing period.

(E) Left, the duration of gaze on landscape scene images was quantified for 1 s during the free-viewing period. Middle, the duration of gaze on the central zone was quantified for 450 ms after scene onset. Right, the distance of eye movements was quantified for 1 s during the free-viewing period. Statistical differences between groups were analyzed using repeated measures one-way ANOVA (scene gaze duration, $F(2.008, 142.6) = 22.01$, $P < 0.0001$; center gaze duration, $F(2.653, 188.3) = 49.76$, $P < 0.0001$), followed by Holm-Šídák's multiple comparisons test ($^{****}P < 0.0001$, $n = 72$).

(F, G) The average firing rates of LHb and PAG in response to landscape scene onset.

(H) The average firing rates of LHb and PAG neurons in response to the landscape scene onset. The LHb response was quantified during the 150–600 ms after object onset. The PAG response was quantified during the 250–800 ms after object onset. Statistical differences between groups were analyzed using repeated measures one-way ANOVA (LHb, $F(1.273, 42.01) = 42.95$, $P < 0.0001$, $n = 34$; PAG, $F(1.862, 80.07) = 12.73$, $P < 0.0001$, $n = 44$), followed by Holm-Šídák's multiple comparisons test ($^{**}P < 0.01$, $^{****}P < 0.0001$). Data are presented as mean ± SEM.

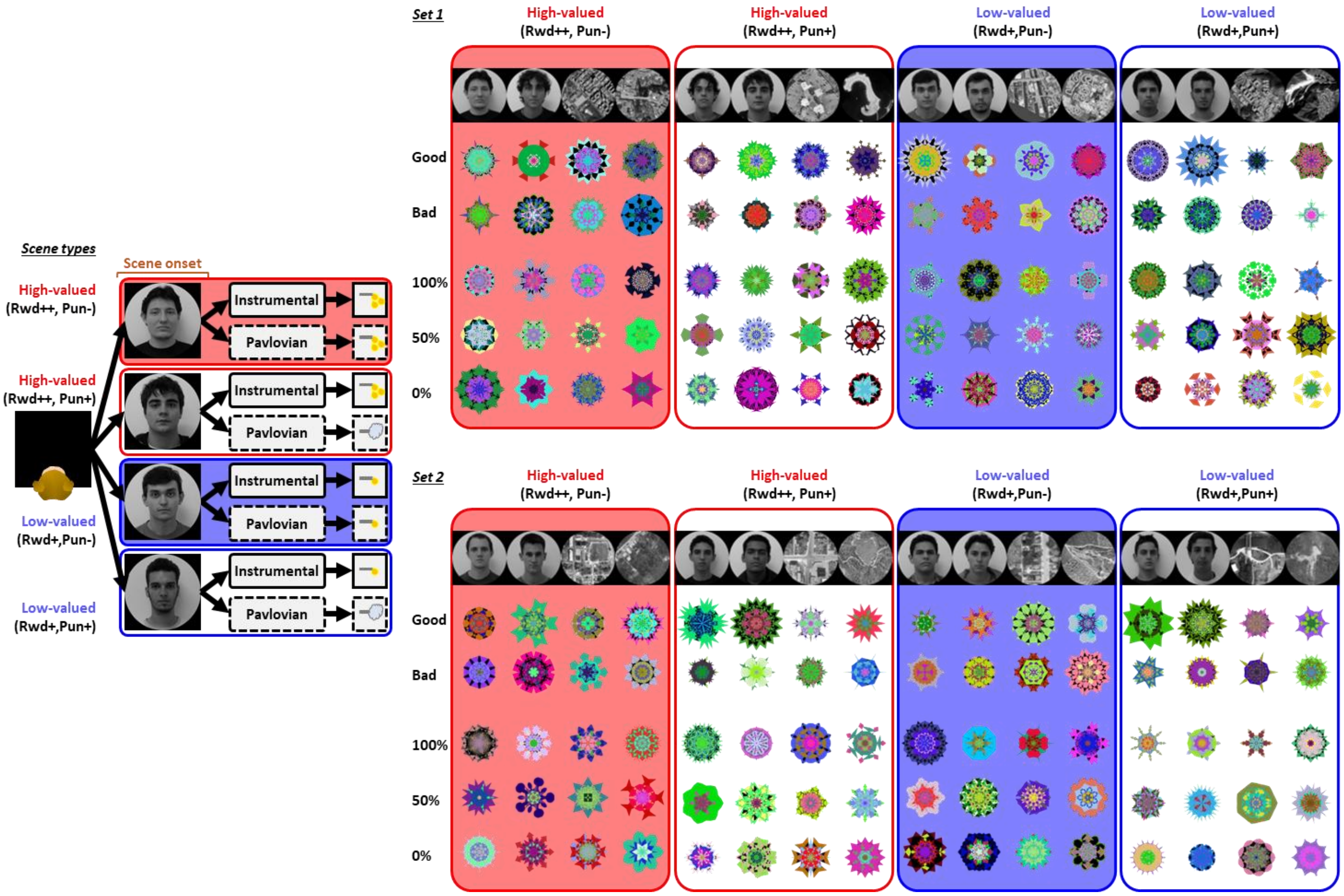

Figure S3. Stimulus sets used in the instrumental and Pavlovian tasks

The figure displays examples of all scene categories and their corresponding object sets used in the two stimulus sets (Set 1 and Set 2). A total of 16 background scene images were used in each set, comprising four context categories that differed in reward and punishment outcomes: high-value reward without punishment (Rwd++ Pun–), high-value reward with punishment (Rwd++ Pun+), low-value reward without punishment (Rwd+ Pun–), and low-value reward with punishment (Rwd+ Pun+). Each context included four distinct scenes (two face scenes and two landscape scenes). Every scene image was paired with a unique set of five associated objects: one good and one bad object for the instrumental task, and three additional objects signaling 100%, 50%, and 0% reward or punishment probabilities for the Pavlovian task. In total, 80 objects were linked to the 16 scenes and were presented across 384 trials in a single block. Two independent stimulus sets were tested in each monkey, with one set (Set 2) having the reward outcomes reversed across monkeys (i.e., high-valued scenes in one set corresponded to low-valued scenes in the other).

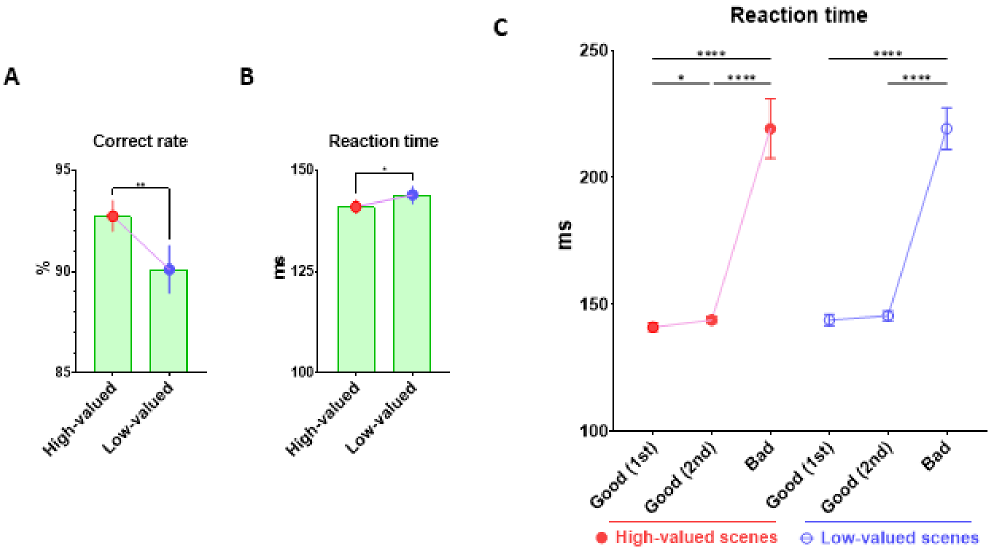

Figure S4. Behavioral measure to assess value in instrumental task

(A) Rate of correct operant responses for high-valued and low-valued scenes. Monkeys exhibited a higher correct response rate in high-valued scenes, where a larger reward was predicted, compared to low-valued scenes (high-valued, 92.74 ± 0.7542; low-valued, 90.10 ± 1.170; Wilcoxon matched-pairs signed rank test; $^{**}P < 0.01$, $n = 72$).

(B) Reaction times for saccades toward the good object in high-valued versus low-valued scenes. Reaction times were faster in high-valued scenes, consistent with higher expected reward (high-valued, 141.0 ± 1.705; low-valued, 143.9 ± 2.202; Wilcoxon matched-pairs signed rank test; $^{*}P < 0.05$, $n = 72$).

(C) Reaction times for the first good object, second good object, and bad object in high-valued versus low-valued scenes. In high-valued scenes, the presentation of a bad object increased the reaction time for the subsequent good object, reflecting additional temporal and effort costs. Statistical differences between groups were analyzed using repeated measures one-way ANOVA ($F(1.724, 122.4) = 49.27$, $P < 0.0001$, $n = 72$), followed by Holm-Šídák's multiple comparisons test ($^{*}P < 0.05$, $^{****}P < 0.0001$). Data are presented as mean ± SEM.

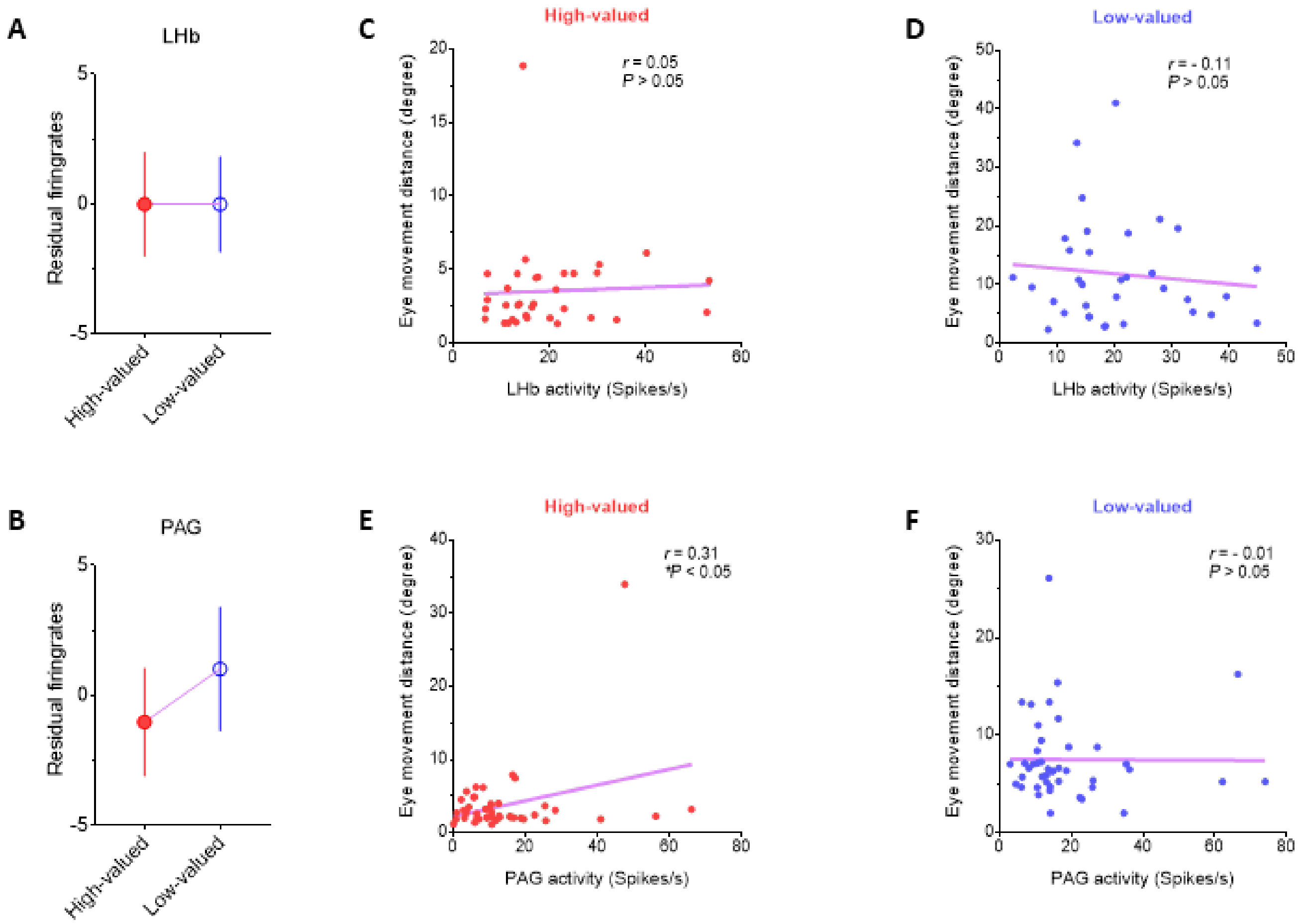

Figure S5. Correlation between neuronal activity and inhibitory control of eye movements in high- and low-valued scenes

(A-B) Residual firing rates of LHb and PAG neurons after regressing out the effect of eye movement distance. Firing rates and eye movement distance were quantified during the −200 to 0 ms period before object onset, as shown in Figures 6A and C (LHb: high-valued, $8.824 \times 10^{-7} \pm 1.997$; low-valued, $- 5.882 \times 10^{-7} \pm 1.824$, Wilcoxon matched-pairs signed rank test; $P > 0.05$, $n = 34$; PAG: high-valued, $- 1.019 \pm 2.060$; low-valued, 1.019 ± 2.372, Wilcoxon matched-pairs signed rank test; $P > 0.05$, $n = 44$).

(C–D) Correlation between the average firing rates of LHb neurons (from Figure 6B) and the average eye movement distances (from Figure 6A) in high- and low-valued scenes (Pearson correlation analysis, high-valued, $r = 0.05$, $P > 0.05$; low-valued, $r = - 0.11$, $P > 0.05$, $n = 34$).

(E–F) Correlation between the average firing rates of PAG neurons (from Figure 6C) and the average eye movement distances (from Figure 6A) (Pearson correlation analysis, high-valued, $r = 0.31$, $^{*}P < 0.05$; low-valued, $r = - 0.01$, $P > 0.05$, $n = 44$).
